# Efficacy and safety of different anti-VEGF agents combined with pars plana vitrectomy in proliferative diabetic retinopathy: a systematic review and network meta-analysis of randomized controlled trials

**DOI:** 10.3389/fendo.2026.1772351

**Published:** 2026-03-25

**Authors:** Songjie Lu, Yitong Lin, Lan Lin, Sheng Chen

**Affiliations:** 1 Fujian University of Traditional Chinese Medicine, Fuzhou, China; 2 Department of Ophthalmology, The Second Affiliated Hospital of Fujian University of Traditional Chinese Medicine, Fuzhou, China

**Keywords:** proliferative diabetic retinopathy, anti-vascular endothelial growth factor agents, pars plana vitrectomy, efficacy, safety, network meta-analysis

## Abstract

**Objective:**

To compare the efficacy and safety of different anti-vascular endothelial growth factor (anti-VEGF) agents in combination with pars plana vitrectomy (PPV) for the treatment of proliferative diabetic retinopathy (PDR), in order to guide clinical decision-making.

**Methods:**

A systematic search was conducted in PubMed, EMBASE, the Cochrane Library, and Web of Science for relevant randomized controlled trials (RCTs) up to November 1, 2025. The outcomes assessed included intraoperative bleeding rate, postoperative vitreous hemorrhage rate, changes in best-corrected visual acuity (BCVA), incidence of iatrogenic retinal breaks, reoperation rate, and the duration of surgery. A network meta-analysis was performed to evaluate the efficacy and safety of bevacizumab (IVB), ranibizumab (IVR), conbercept (IVC), and aflibercept (IVA) in combination with PPV. The risk of bias was assessed using the RoB 2 tool, and the quality of evidence was graded using the GRADE framework. This study was registered with the Prospective Register of Systematic Reviews (CRD420251251306).

**Results:**

A total of 22 RCTs involving 1, 388 patients (1, 416 eyes) and five treatment regimens were included. Compared with PPV alone, all anti-VEGF agents significantly reduced the risk of intraoperative bleeding, as indicated by 95% confidence intervals (CIs) not crossing the null value of 1. IVR demonstrated the most favorable outcome (OR = 0.03, 95% CI: 0–0.44; SUCRA = 86.1%). For preventing postoperative vitreous hemorrhage, IVA was the most effective (OR = 0.19, 95% CI: 0.05–0.66; SUCRA = 73%), followed by IVC (OR = 0.25, 95% CI: 0.13–0.49) and IVB (OR = 0.30, 95% CI: 0.17–0.53). Regarding postoperative visual improvement, IVC showed the most favorable trend (MD=-0.72, 95% CI: -1.64 to 0.20; SUCRA = 85.2%). IVB significantly shortened the duration of surgery (MD=-19.96, 95% CI: -29.09 to -10.82; SUCRA = 93.5%) and reduced reoperation rate (OR = 0.36, 95% CI: 0.21–0.63; SUCRA = 90.8%). IVR was the most effective in reducing iatrogenic retinal breaks (OR = 0.08, 95% CI: 0.01–0.52; SUCRA = 83.9%).

**Conclusions:**

Different anti-VEGF agents demonstrate distinct advantages during the perioperative period of PDR. IVR and IVC excel in controlling intraoperative and postoperative bleeding, IVB is the most efficient in improving surgical efficiency, and IVC shows the greatest potential for vision improvement. Clinical decisions should be based on individualized treatment goals.

**Systematic Review Registration:**

https://www.crd.york.ac.uk/PROSPERO/view/CRD420251251306, identifier CRD420251251306.

## 1 Introduction

The prevalence of diabetes is steadily increasing, presenting a substantial global public health challenge. By 2045, it is projected that the number of individuals living with diabetes will approach 700 million (1). Diabetic retinopathy (DR) is one of the most prevalent and visually debilitating microvascular complications of diabetes. The global incidence of DR is expected to rise from 103 million in 2020 to 160 million by 2045, marking an increase of over 55% (2). The burden of PDR is particularly striking in real-world clinical settings. For instance, a national study from Bhutan reported that among diabetic patients presenting for vitreoretinal consultation for the first time, the prevalence of PDR was 12.5%, highlighting the substantial proportion of patients who already have advanced, vision-threatening disease at the point of specialist referral (3). Proliferative diabetic retinopathy (PDR) represents the advanced stage of this condition, primarily characterized by ischemia-driven neovascularization and fibrovascular proliferation. Clinically, PDR is frequently associated with vitreous hemorrhage and tractional retinal detachment, significantly heightening the risk of irreversible vision loss (4). For patients with PDR who experience persistent vitreous hemorrhage or difficult-to-resolve tractional retinal changes, pars plana vitrectomy (PPV) remains a vital salvage procedure. PPV effectively clears vitreous opacity, alleviates vitreoretinal traction, and addresses proliferative membranes (5). Nevertheless, despite ongoing advancements in minimally invasive instrumentation and surgical techniques, complications such as intraoperative bleeding, iatrogenic retinal breaks, and postoperative vitreous hemorrhage remain relatively frequent (6–8). These complications can lead to extended surgery durations, increased procedural complexity, compromised anatomical reattachment, and elevated reoperation rates, all of which contribute to patient burden and adversely affect long-term outcomes (9).

The introduction of anti-vascular endothelial growth factor (anti-VEGF) therapy has emerged as a novel intervention for mitigating perioperative risks in PDR. Several randomized controlled trials have indicated that combining anti-VEGF therapy with PPV both pre- and post-operatively can expedite the regression of abnormal neovascularization, reduce intraoperative active bleeding, enhance the feasibility of membrane separation, and potentially decrease the risk of early postoperative hemorrhage and other related complications (10–13). The anti-VEGF agents commonly utilized in clinical practice include bevacizumab (10), ranibizumab (11), conbercept (12), and aflibercept (13). Prior meta-analyses have demonstrated that preoperative anti-VEGF treatment in PDR patients facilitates smoother surgical procedures, promotes visual recovery, and may reduce the risk of early postoperative vitreous hemorrhage and retinal detachment (14, 15). Traditional meta-analyses have consistently shown that perioperative anti-VEGF pretreatment generally improves intraoperative parameters and specific postoperative outcomes (16, 17). However, due to the absence of sufficient head-to-head trials comparing different anti-VEGF agents, existing evidence remains inconclusive regarding which specific anti-VEGF agent, when combined with PPV, offers superior efficacy and safety. To address this gap, the current study aimed to incorporate randomized controlled trials and utilize systematic review and network meta-analysis methodologies to comprehensively compare and rank the efficacy and safety of various anti-VEGF agents in conjunction with PPV, thereby providing higher-quality evidence to inform personalized perioperative medication decisions for PDR.

## 2 Materials and methods

This network meta-analysis (NMA) follows the guidelines outlined in the Preferred Reporting Items for Systematic Reviews and Meta-Analyses extension for network meta-analyses (**Supplementary Table 1**) (18). Due to the absence of direct head-to-head randomized controlled trials comparing various anti-VEGF treatment regimens, the NMA incorporates both direct and indirect evidence within a unified control framework to estimate the relative effects of different anti-VEGF agents in combination with PPV in terms of efficacy and safety. Furthermore, it assesses the probability ranking of each treatment regimen (19). To ensure transparency, robustness, and innovation, the protocol for this study has been registered with the Prospective Register of Systematic Reviews (CRD420251251306).

### 2.1 Data sources and search strategy

A systematic search was conducted across the PubMed, EMBASE, Cochrane Library, and Web of Science databases. The search terms included *diabetic retinopathy*, *proliferative diabetic retinopathy*, *randomized clinical trial*, *Bevacizumab*, *Ranibizumab*, *Conbercept*, *Aflibercept*, *anti-vascular endothelial growth factor*, *pars plana vitrectomy*, and *microincision vitrectomy surgery*. The search encompassed literature from the inception of each database through November 1, 2025, and utilized a combination of free-text terms and subject headings, with no restrictions on language. Detailed search strategies for each database are outlined in **Supplementary Table 2**.

### 2.2 Selection criteria

Inclusion Criteria:

1. Participants were patients clinically diagnosed with PDR who underwent PPV for surgical indications such as vitreous hemorrhage or tractional retinal detachment, within randomized controlled trials (RCTs).
2. Intervention: The experimental group received PPV combined with anti-VEGF therapy, while the control group underwent either PPV alone or PPV in combination with an alternative anti-VEGF agent.
3. RCTs must report at least one of the following outcome measures:

Intraoperative outcomes: Number of eyes with intraoperative bleeding, number of iatrogenic retinal breaks, and the duration of surgery.

Postoperative outcomes: Number of eyes with postoperative vitreous hemorrhage, best-corrected visual acuity (BCVA, expressed in LogMAR or ETDRS letter score), and number of eyes requiring reoperation.

Exclusion Criteria:

1. Non-randomized controlled trials, retrospective studies, case reports, reviews, or conference abstracts.
2. Studies including participants with vitreoretinal diseases not attributable to PDR, or those that do not allow for the extraction of PDR-specific subgroup data.
3. Studies that do not report the specified intraoperative or postoperative outcomes, or where data could not be extracted due to the merging of studies.
4. Studies where the full text is unavailable, or those with duplicate publications, overlapping data sources, or insufficient information.

Prior to inclusion, studies were screened based on titles and abstracts. All selected RCTs were independently verified by two reviewers to ensure they represented the most recent published evidence.

### 2.3 Data extraction and quality assessment

The data were independently extracted by the researchers from the RCTs in accordance with the Preferred Reporting Items for Systematic Reviews and Meta-Analysis (PRISMA) guidelines. Any discrepancies encountered were resolved through discussion with the second author. The following data were extracted from each study: the first author, publication year, sample size, baseline patient characteristics (age, gender), study location, follow-up duration, and the specific intervention protocols for both the experimental and control groups (including details on anti-VEGF agents, timing of administration, and dosages). For continuous outcomes, the primary effect measure was the change in BCVA, with particular emphasis on extracting the mean change values and standard deviations from baseline to the follow-up endpoint. In cases where only the baseline and endpoint BCVA values were reported, the mean change and standard deviation were calculated using the following formula:


MEANchange=Endpoint Mean−Baseline Mean

$$SDchange=\sqrt{{\text{Baseline SD}}^{2}+{\text{Endpoint SD}}^{2}-2R\cdot \text{Baseline SD}\cdot \text{Endpoint SD}}$$

In this formula, R represents the correlation coefficient between baseline and endpoint BCVA. In instances where the correlation coefficient is either not reported or cannot be inferred, this study assumes a value of R = 0.5 for the conversion, with a sensitivity analysis conducted to evaluate the robustness of the results.

The duration of surgery is recorded as a continuous variable for each procedure, excluding any changes from baseline to follow-up. Consequently, the mean and standard deviation of surgery duration for each group are directly extracted. For binary outcomes, both the number of events and the total sample size (either by eye or by patient, as defined in the original studies) are recorded for each group, and these values are subsequently used in effect size aggregation analyses.

To assess potential heterogeneity arising from variations in outcome definitions across studies, we systematically extracted the criteria used for each reported outcome, including intraoperative bleeding, postoperative vitreous hemorrhage, and reoperation. Although most studies employed clinically comparable definitions (e.g., intraoperative bleeding graded by severity, postoperative vitreous hemorrhage assessed using fundus visibility scales, and reoperation defined as repeat vitrectomy for recurrent hemorrhage or retinal detachment), minor differences were noted. A detailed summary of these definitions is provided in **Supplementary Table 3**. The impact of these variations on the pooled estimates was evaluated through sensitivity analyses, as described below.

The methodological quality of the included RCTs was evaluated using the Cochrane Risk of Bias tool, RoB 2 (20). In accordance with the principles of evidence-based medicine, RoB 2 identifies potential sources of bias that may influence effect estimates across the following five domains:

1. Randomization process (bias arising from randomization): Assesses the adequacy of random sequence generation and allocation concealment.
2. Deviations from intended interventions (bias due to deviations from intended interventions): Evaluates adherence to the intervention and the implementation of blinding.
3. Missing outcome data (bias due to missing outcome data): Analyzes dropout rates and missing data, along with their potential impact on the results.
4. Outcome measurement bias (bias in the measurement of outcomes): Examines whether outcome measures were objective, consistent, and assessed with blinding.
5. Selection reporting bias (bias in the selection of reported results): Identifies risks of selectively reporting significant results or omitting essential data.

The risk levels for each domain are classified as *low risk*, *some concerns*, or *high risk*. Two researchers independently performed the assessment, and any discrepancies were resolved through discussion or adjudication by a third reviewer.

### 2.4 Statistical analysis

This study employed Stata 17.0 MP for the network meta-analysis. For continuous outcome variables, where studies used the same scale and units, the mean deviation (MD) and its 95% confidence interval (CI) were calculated. In instances where different scales or units were employed across studies for the same outcome, the standardized mean deviation (SMD) and its 95% CI were used. For binary outcomes, the odds ratio (OR) and its corresponding 95% CI were applied. In multi-arm trials, an augmented format was utilized to generate pairwise comparisons while preserving the within-study correlation structure, thereby mitigating the risk of underestimating standard errors due to the repeated use of control groups. For binary outcomes with zero events or zero non-events, continuity correction (r = r + 0.5, n = n + 1) was applied to all treatment arms within the study prior to effect size calculation to prevent infinite OR estimates.

The primary analysis was conducted assuming consistency, using a random-effects model to pool results, with between-study variance (τ²) estimated via restricted maximum likelihood (REML). In the presence of closed loops within the network, a global inconsistency test was performed to assess consistency, and a local inconsistency test using the node-splitting method was employed. A p-value < 0.1 was considered indicative of potential inconsistency. The loop inconsistency factor (IF, on the log effect scale) was utilized to evaluate consistency within closed loops. A 95% CI that included 0 suggested no statistical evidence of inconsistency between direct and indirect evidence.

To further explore heterogeneity in direct treatment comparisons, the *network convert pair* command was used to extract all pairwise comparisons from the NMA dataset. Random-effects meta-regression was then performed for each direct comparison to estimate the degree of heterogeneity between studies. A network diagram was generated to visualize the geometry of the treatment network, where the size of each node represented the total sample size for each treatment, and the thickness of the connecting lines indicated the number of studies contributing to each direct comparison.

For treatment ranking, multiple ranking metrics were utilized, including the surface under the cumulative ranking curve (SUCRA), the probability of being the best treatment (PreBest), and the average rank, to enhance the robustness and interpretability of the results. Publication bias and small-sample effects were assessed using funnel plots with comparison-based corrections. Sensitivity analysis was conducted using a leave-one-out method, in which each study was sequentially excluded, and the random-effects consistency model was re-applied to compare the direction and magnitude of pooled effects. Additionally, univariate network meta-regression was performed to explore the influence of study-level covariates on treatment effects, with regression coefficients, 95% CIs, and p-values from the Wald test reported. Statistical significance was defined as p < 0.05, indicating a potential modifying effect of the covariate.

To further explore whether the injection-to-surgery interval influenced treatment effects, we performed network meta-regression incorporating the timing of preoperative anti-VEGF administration as a continuous covariate (**Supplementary Tables 4.13–4.18**). The interval was defined as the number of days between injection and vitrectomy; for studies reporting a range, the midpoint was used. Postoperative injections were excluded from this analysis.

### 2.5 GRADE assessment of evidence certainty

The certainty of the evidence from the NMA was evaluated using the GRADE framework and CINeMA (Confidence in Network Meta-analysis) tool. Initially, RCTs were classified as having *high certainty*. The assessment of evidence certainty was conducted across six domains: risk of bias within studies, indirectness, imprecision, heterogeneity, inconsistency, and risk of bias across studies (publication bias and small sample effects).

The risk of bias within individual studies was evaluated for each domain using the RoB 2 tool. The contribution of bias to the network estimates was then weighted and aggregated at the comparison level using the CINeMA contribution matrix. Indirectness was assessed based on the assumptions of transitivity and exchangeability, considering predefined potential effect modifiers, such as baseline severity, intervention intensity, and follow-up duration. The consistency between direct and indirect evidence was examined with respect to the population, intervention, comparator, and outcome measures.

Imprecision was evaluated using predefined minimum clinically important difference (MID). For binary outcomes, an OR of 1.25 was set as the threshold for clinical significance, while for continuous outcomes, a SMD of 0.5 was used. Evidence was considered imprecise if the 95% confidence interval crossed the null value or the specified MID threshold.

Heterogeneity was primarily assessed using the between-study variance (τ²) derived from the random-effects model, along with the position of the prediction interval relative to the MID. In networks with closed loops, inconsistency was evaluated through the CINeMA tool’s built-in assessment of direct and indirect consistency.

Risk of bias across studies was assessed by reviewing trial registration, conducting a grey literature search, and evaluating small sample effects using comparison-adjusted funnel plots. Each domain was rated as *no concerns*, *some concerns*, or *serious concerns*, with evidence certainty downgraded by one level for *some concerns* and two levels for *serious concerns*, in accordance with GRADE principles. The final certainty of evidence was categorized as *high*, *moderate*, *low*, or *very low*.

## 3 Results

### 3.1 Systematic review and characteristics of the included studies

**Figure 1:**
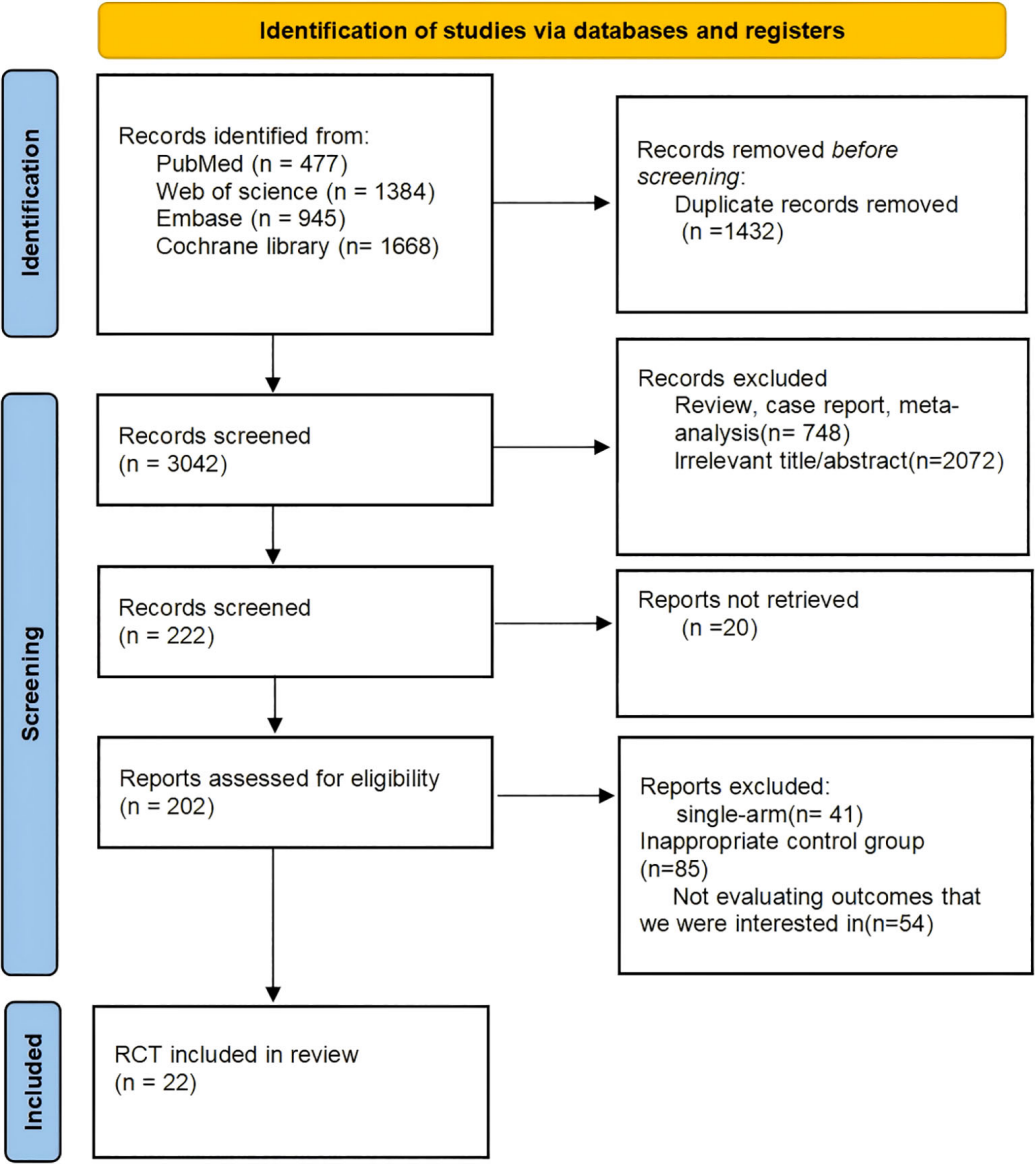
Flow diagram of the literature search and selection process. This study follows the evidence-based PRISMA guidelines.

A comprehensive literature search initially yielded 4, 474 records from four major databases, based on a predefined search strategy. Following abstract screening to exclude duplicates and irrelevant articles, 202 studies were deemed eligible for full-text evaluation. Ultimately, 22 studies met the inclusion criteria (**Figure 1**), encompassing a total of 1, 388 patients (1, 416 eyes) who underwent one of five treatment regimens: pars plana vitrectomy (PPV), PPV combined with intravitreal bevacizumab (PPV-IVB), PPV combined with intravitreal conbercept (PPV-IVC), PPV combined with intravitreal ranibizumab (PPV-IVR), and PPV combined with intravitreal aflibercept (PPV-IVA).

The analysis comprised 22 RCTs, with multicenter data from across the globe, including China (8 studies), Iran (3 studies), Italy (2 studies), and other countries. The total sample size was moderate, with a predominant representation of middle-aged and older individuals (mean age ranging from 48 to 60 years) and a generally balanced gender distribution. All control groups received PPV alone, while the experimental groups were treated with anti-VEGF agents administered 1 to 20 days prior to surgery. bevacizumab (IVB) was the most frequently employed drug (12 studies), followed by conbercept (IVC), ranibizumab (IVR), and aflibercept (IVA). Postoperative follow-up durations varied, with 3 to 12 months being the most common range. Overall, the included studies were representative and comparable in terms of patient demographics, intervention protocols, and follow-up design. Comprehensive details for all included studies are presented in **Table 1** and **Supplementary Table 4**.

**Table 1:** Characteristics of included studies.

No.	First Author & Publication Year	Sample Size (Exp./Ctrl.)	Age	Gender Ratio (M/F)	Country	Follow-up Time	Intervention in Experimental Group	Intervention in Control Group
1	Arevalo JF 2019 (10)	102/112	59.5/61.3	123/91	Global	12m	IVB 1.25 mg 3–5 days before PPV	PPV
2	El-Batarny AM 2008 (21)	15/15	44/46	/	Oman	7-18m	IVB 1.25 mg 5–7 days before PPV	PPV
3	Comyn O 2017 (11)	15/15	57.1/48.7	12/18	UK	3m	IVR 0.5 mg 7 days before PPV	PPV
4	Ahn J 2011 (22)	36/34	51/55	39/31	South Korea	6m	IVB 1.25 mg 1–14 days before PPV	PPV
5	Yang X 2016 (12)	54/53	48.6/49.6	51/37	China	3m	IVC 0.5 mg 3 days before PPV	PPV
6	Su L 2016 (23)	18/18	/	/	China	3m	IVC 0.5 mg 7 days before PPV	PPV
7	Hernández-Da MSE 2010 (24)	20/20	55.7/55.7	19/21	Mexico	6m	IVB 1.25 mg 2 days before PPV	PPV
8	Farahvash MS 2011 (25)	18/17	58.7/58.3	18/17	Iran	8m	IVB 1.25 mg 5–12 days before PPV	PPV
9	Ahmadieh H 2009 (26)	35/33	56.7/53.7	34/34	Iran	1m	IVB 1.25 mg 7 days before PPV	PPV
10	di Lauro R 2010 (27)	48/24	/	/	Italy	6m	IVB 1.25 mg 7 or 20 days before PPV	PPV
11	Faisal SM 2018 (28)	28/28	58.1/57.2	26/30	Pakistan	>3m	IVB 1.25 mg 7 days before PPV	PPV
12	Li S 2022 (29)	32/16	48.4/53.1	27/21	China	>3m	IVR 0.5 mg 1 or 3 days before PPV	PPV
13	Manabe A 2015 (30)	32/34	59.9/59.2	52/10	Japan	1m	IVB 0.16 mg 1 day before PPV	PPV
14	Modarres M 2009 (31)	22/18	55.8/53.2	/	Iran	7m	IVB 2.5 mg 3–5 days before PPV	PPV
15	Rizzo S 2008 (32)	11/11	52/52	/	Italy	6m	IVB 1.25 mg 5–7 days before PPV	PPV
16	Sohn EH 2012 (33)	10/10	52/52	12/7	USA	3m	IVB 1.25 mg 3–7 days before PPV	PPV
17	Zaman Y 2013 (34)	30/24	52/52	32/22	Pakistan	6m	IVB 1.25 mg 7 days before PPV	PPV
18	Ding Y 2023 (35)	124/28	50.6/55.2	60/92	China	>3m	IVC 0.5 mg 7 days before PPV	PPV
19	Qu JF 2023 (13)	64/64	52.9/55.3	82/46	China	3m	IVA 0.5 mg 1–5 days before PPV	PPV
20	Yang Z 2023 (36)	36/12	50.2/55.3	25/23	China	3d	IVC 0.5 mg 3 or 7 or 14 days before PPV	PPV
21	Jiang T 2020 (37)	15/15	55.5/53.5	16/14	China	12m	IVC 0.5 mg immediately after PPV	PPV
22	Ren X 2019 (38)	25/25	57/62	30/20	China	6m	IVC 0.5 mg immediately after PPV	PPV

Exp., experimental group; Ctrl., control group; IVB, Bevacizumab; IVR, Ranibizumab; IVC, Conbercept; IVA, Aflibercept; PPV, Pars Plana Vitrectomy; M, male; F, female.

To further assess the transitivity assumption underlying the network meta-analysis, we examined the distribution of key clinical characteristics and potential effect modifiers across treatment arms in the included studies (**Table 2**). Overall, baseline disease severity appeared comparable among the different intervention groups. The proportion of eyes with tractional retinal detachment (TRD) ranged from 0% to 100% across studies, reflecting the variability in inclusion criteria, but within each study, TRD rates were generally balanced between the anti-VEGF and control groups. Similarly, the proportion of eyes with vitreous hemorrhage (VH) was consistently high and evenly distributed across treatment arms in most studies, consistent with the surgical indications for PDR.

**Table 2:** Distribution of potential effect modifiers across included studies.

No.	First Author & Publication Year	Sample Size (Exp./Ctrl.)	TRD (%)	VH (%)	Pseudophakic (%)	Prior PRP (%)	Mean HbAlc (%)	Diabetes Duration (years)
1	Arevalo JF 2019 (10)	102/112	100/100	53/56	28.4/24.0	77.4/82.1	8.5/8.9	/
2	El-Batarny AM 2008 (21)	15/15	26.7/26.7	100/100	/	86.7/93.3	/	/
3	Comyn O 2017 (11)	15/15	100/100	60/80	33.3/20.0	100/100	8.2/9.3	19/21
4	Ahn J 2011 (22)	36/34	11.1/11.8	58.3/73.5	8.3/5.9	61.6/64.7	8.1/8.1	/
5	Yang X 2016 (12)	54/53	9.3/11.3	40.7/43.4	20.4/15.1	20.4/30.2	7.9/7.6	16.7/15.9
6	Su L 2016 (23)	18/18	100/100	100/100	/	/	/	/
7	Hernández-Da MSE 2010 (24)	20/20	100/100	0/0	0/0	/	/	/
8	Farahvash MS 2011 (25)	18/17	34.3/34.3	100/100	5.6/11.8	44.4/47.1	8.4/8.3	13.5/10.1
9	Ahmadieh H 2009 (26)	35/33	18.6/21.2	80.0/78.8	/	80.0/81.8	/	/
10	di Lauro R 2010 (27)	48/24	100/100	100/100	/	/	/	/
11	Faisal SM 2018 (28)	28/28	/	100/100	/	/	/	6.4/7.4
12	Li S 2022 (29)	32/16	71.9/81.3	100/100	/	/	/	/
13	Manabe A 2015 (30)	32/34	1.5/1.2	90.6/88.2	12.5/11.8	71.9/67.6	8.0/7.6	/
14	Modarres M 2009 (31)	22/18	100/100	/	0/0	/	/	/
15	Rizzo S 2008 (32)	11/11	100/100	63.6/63.6	/	/	/	/
16	Sohn EH 2012 (33)	10/10	100/100	50/50	/	100/100	/	21/21
17	Zaman Y 2013 (34)	30/24	100/100	100/100	/	/	/	/
18	Ding Y 2023 (35)	124/28	33.9/35.7	100/100	/	/	/	9.8/13.0
19	Qu JF 2023 (13)	64/64	15.6/9.4	100/100	4.7/3.1	45.3/39.1	6.9/6.5	8.4/9.0
20	Yang Z 2023 (36)	36/12	/	100/100	/	/	/	12.0/15.3
21	Jiang T 2020 (37)	15/15	100/100	100/100	20.0/6.7	40.0/33.3	6.7/6.8	13.2/9.9
22	Ren X 2019 (38)	25/25	0/0	100/100	24/32	52/68	/	10.4/12.0

Exp., experimental group; Ctrl., control group; TRD, tractional retinal detachment; VH, vitreous hemorrhage; PRP, panretinal photocoagulation; HbAlc, Hemoglobin A1c.

Regarding ocular treatment history, the proportion of pseudophakic eyes and the percentage of patients with prior panretinal photocoagulation (PRP) were also comparable between experimental and control groups within individual studies, although some variation existed across trials. Systemic factors relevant to disease progression and surgical outcomes, including mean hemoglobin A1c (HbA1c) levels and diabetes duration, were similarly distributed between treatment arms in studies that reported these variables. This balanced distribution of baseline demographic, ocular, and systemic characteristics across treatment groups supports the validity of the transitivity assumption and enhances the credibility of the indirect comparisons in this network meta-analysis.

### 3.2 RoB 2 quality assessment

The quality of the included studies was assessed using the RoB 2 tool. Among the 22 studies, 14 were classified as having a low overall risk of bias, while 8 were rated as presenting *some concerns*. The majority of studies performed well in critical areas, such as randomization methods, intervention implementation, data completeness, and outcome measurement, reflecting generally high-quality research. In terms of randomization, most studies employed methods such as computer-generated randomization, random number tables, or sealed envelope techniques for allocation, and reported balanced baseline characteristics. However, a small number of studies merely stated that groups were *randomized* without providing details on the random sequence generation or allocation concealment procedures, introducing a potential risk of selection bias. Regarding the execution of interventions, the majority of studies utilized double-blind or triple-blind designs, with clear separation between surgeons and assessors to ensure standardization of the intervention. A few studies did not explicitly clarify whether blinding was applied to the patients or surgeons, which may have introduced minor bias in the subjective assessment of outcomes. Concerning data completeness, the vast majority of studies reported high treatment completion rates and low dropout rates, with adequate explanations provided for any missing data. A few studies lacked comprehensive follow-up details; however, no systematic data loss or selective exclusions were identified, suggesting good overall data integrity. For outcome measurement, most studies employed objective or standardized indicators, such as surgery duration, vitreous hemorrhage status, and visual acuity (LogMAR/ETDRS) as primary outcomes, with a high level of standardization in the measurement tools used. Several studies explicitly applied blinding during outcome assessment. In terms of reporting, the majority of studies were pre-registered or fully disclosed pre-specified outcomes, with no evident signs of selective reporting. Overall, the risk of bias across domains was relatively low, and the quality of the included studies was deemed satisfactory, providing a strong basis for confidence in the results. Detailed assessment results are presented in **Supplementary Figure 1**.

### 3.3 Network meta-analyses

#### 3.3.1 Consistency and inconsistency assessment

In this study, the primary outcome measures included the intraoperative bleeding rate, postoperative vitreous hemorrhage rate, and changes in BCVA, while the secondary outcomes comprised the intraoperative iatrogenic retinal break rate, reoperation rate, and surgery duration. The evidence networks for each outcome did not form closed loops, precluding the performance of global inconsistency tests, local inconsistency tests, or loop-specific inconsistency analyses.

Consequently, a NMA was conducted under the assumption of consistency using a consistency model. To assess the validity of the transitivity assumption, we compared key clinical characteristics and potential effect modifiers, such as follow-up duration and critical surgical strategies. As detailed in **Table 1**, there were minimal differences in follow-up duration and surgical strategies across the studies, ensuring overall comparability.

#### 3.3.2 Intraoperative bleeding rate

**Figure 2:**
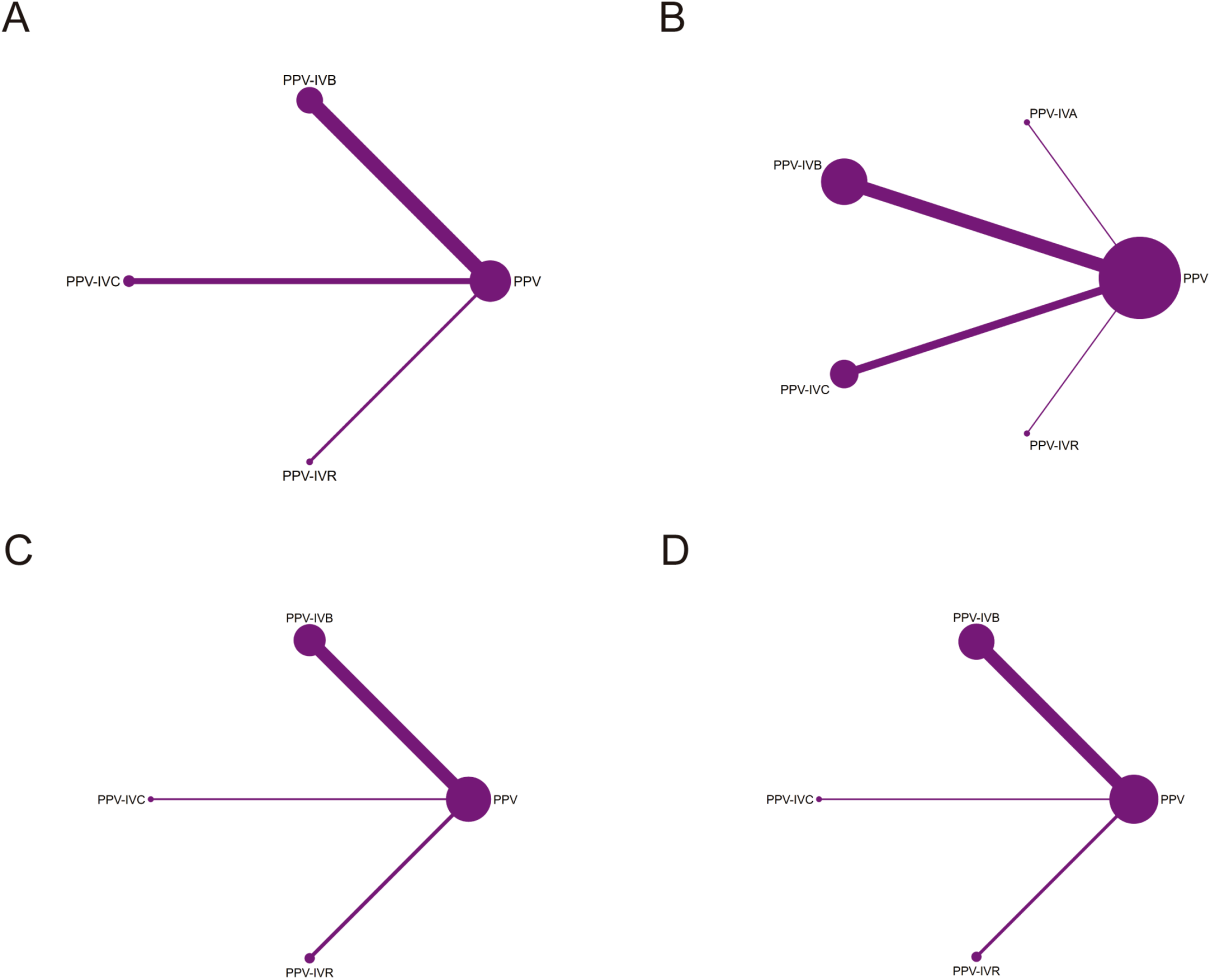
Network diagram comparing PPV with or without intravitreal anti-VEGF injection in patients with PDR. **(A)** Intraoperative bleeding; **(B)** Postoperative vitreous hemorrhage; **(C)** Changes in BCVA; **(D)** Iatrogenic retinal breaks.

**Figure 3:**
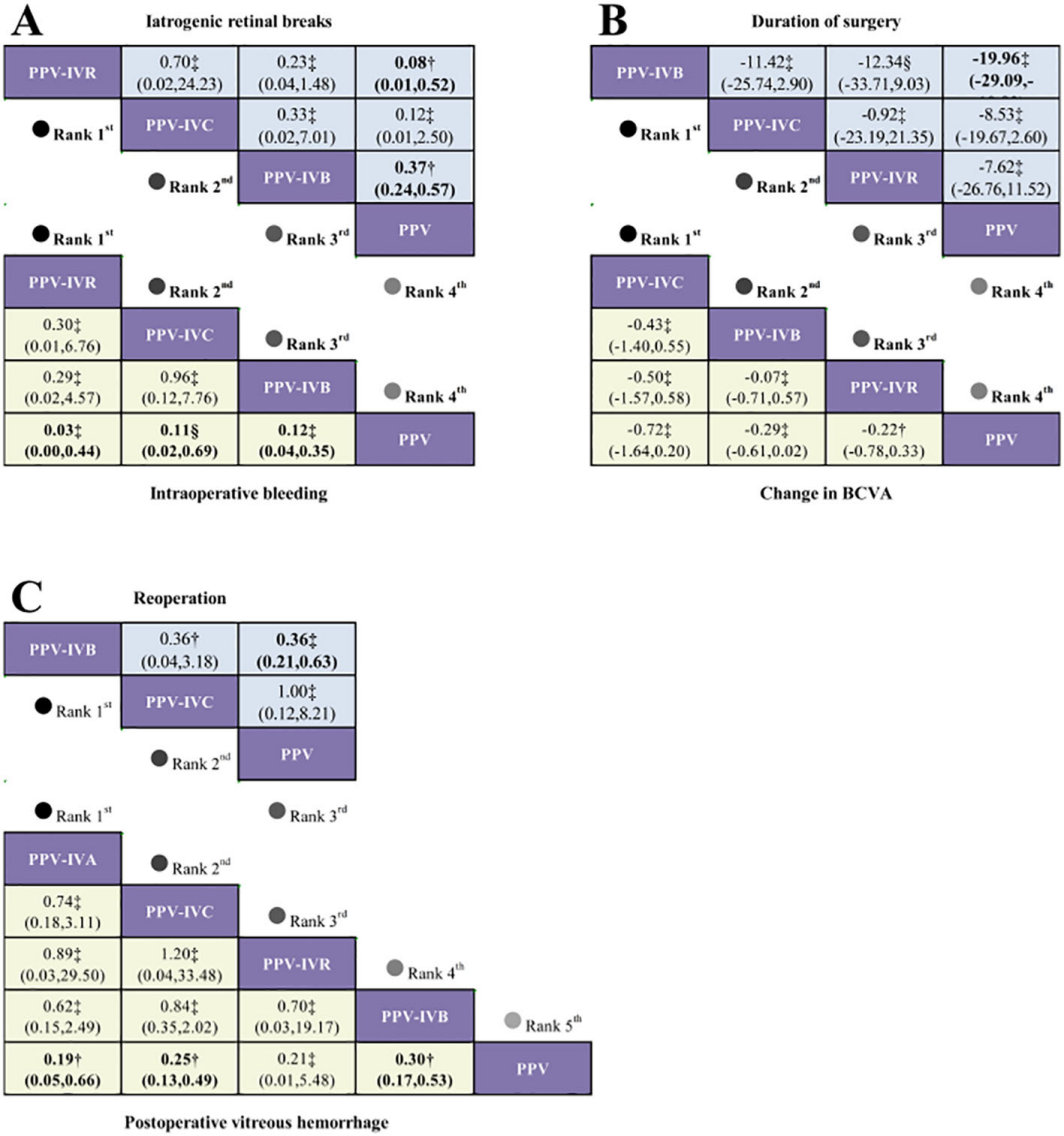
Network diagram comparing the safety and efficacy of PPV alone versus PPV combined with different anti-VEGF agents in patients with PDR. **(A)** Odds ratios (OR) and 95% confidence intervals (CI) for intraoperative bleeding (yellow lower triangular region) and iatrogenic retinal breaks (blue upper triangular region), where OR < 1.00 indicates a lower risk of occurrence. **(B)** Risk ratios and 95% CIs for changes in BCVA (yellow lower triangular region) and duration of surgery (blue upper triangular region), where a mean difference (MD) < 0 indicates a better clinical outcome. **(C)** Odds ratios and 95% CIs for postoperative vitreous hemorrhage (yellow lower triangular region) and reoperation (blue upper triangular region). † = moderate certainty of evidence; ‡ = low certainty of evidence; §= very low certainty of evidence.

**Figure 4:**
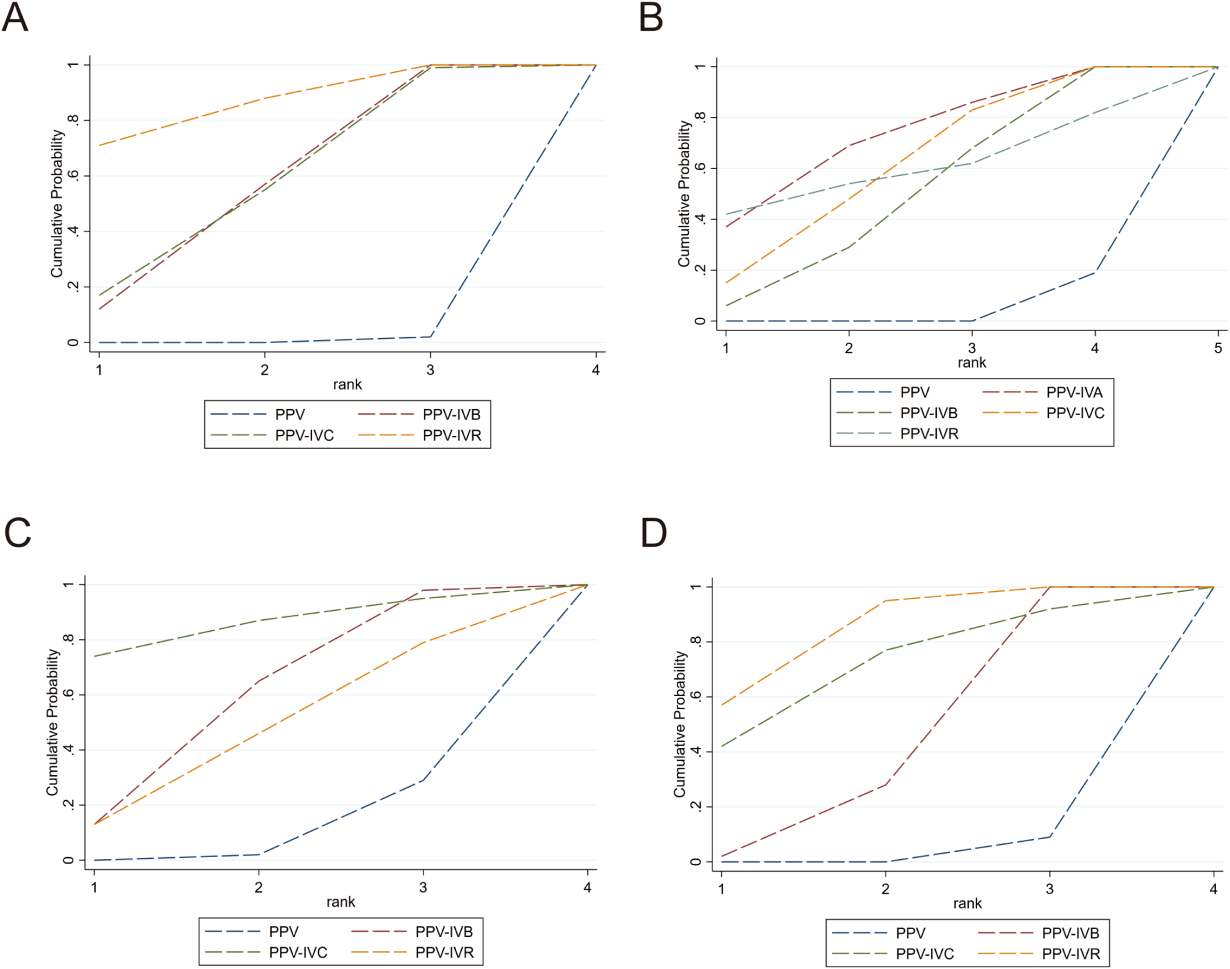
SUCRA ranking curves for the efficacy and safety outcomes of different interventions in PDR patients undergoing PPV. **(A)** Intraoperative bleeding; **(B)** Postoperative vitreous hemorrhage; **(C)** Changes in BCVA; **(D)** Iatrogenic retinal breaks.

A total of 8 studies were included in the analysis of the intraoperative bleeding rate, evaluating four different intervention regimens (see **Figure 2A**). When compared to PPV alone, PPV-IVR (OR = 0.03, 95% CI: 0–0.44), PPV-IVC (OR = 0.11, 95% CI: 0.02–0.69), and PPV-IVB (OR = 0.12, 95% CI: 0.04–0.35) were all associated with a significant reduction in the risk of intraoperative bleeding (see **Figure 3A**). According to the SUCRA ranking, PPV-IVR exhibited the lowest risk of intraoperative bleeding (SUCRA = 86.1%), followed by PPV-IVC (57%) and PPV-IVB (56.3%) (see **Figure 4A**).

#### 3.3.3 Postoperative vitreous hemorrhage rate

A total of 18 studies were included in the analysis of the postoperative vitreous hemorrhage rate, evaluating five different intervention regimens (see **Figure 2B**). When compared to PPV alone, PPV-IVC (OR = 0.25, 95% CI: 0.13–0.49), PPV-IVB (OR = 0.30, 95% CI: 0.17–0.53), and PPV-IVA (OR = 0.19, 95% CI: 0.05–0.66) were all significantly associated with a reduced incidence of postoperative vitreous hemorrhage. PPV-IVR (OR = 0.21, 95% CI: 0.01–5.48) also showed a reduction in the incidence of postoperative vitreous hemorrhage; however, this difference was not statistically significant (see **Figure 3C**). According to the SUCRA ranking, PPV-IVA was associated with the lowest incidence of postoperative vitreous hemorrhage (SUCRA = 73%), followed by PPV-IVC (61.5%), PPV-IVR (60.1%), and PPV-IVB (50.7%) (see **Figure 4B**).

#### 3.3.4 Changes in BCVA

A total of 10 studies were included in the analysis of changes in BCVA, evaluating four intervention regimens (see **Figure 2C**). When compared to PPV alone, PPV-IVC (MD = -0.72, 95% CI: -1.64 to 0.20), PPV-IVB (MD = -0.29, 95% CI: -0.61 to 0.02), and PPV-IVR (MD = -0.22, 95% CI: -0.78 to 0.33) all demonstrated improvements in BCVA, although these differences were not statistically significant (see **Figure 3B**). According to the SUCRA ranking, PPV-IVC showed the greatest improvement in BCVA (SUCRA = 85.2%), followed by PPV-IVB (58.5%) and PPV-IVR (45.8%) (see **Figure 4C**).

#### 3.3.5 Incidence of iatrogenic retinal breaks

Eleven studies were included in the analysis of the incidence of iatrogenic retinal breaks, evaluating four intervention regimens (see **Figure 2D**). When compared to PPV alone, PPV-IVB (OR = 0.37, 95% CI: 0.24–0.57) and PPV-IVR (OR = 0.08, 95% CI: 0.01–0.52) significantly reduced the incidence of iatrogenic retinal breaks. PPV-IVC (OR = 0.12, 95% CI: 0.01–2.50) also reduced the incidence of retinal breaks, though this difference was not statistically significant (see **Figure 3A**). According to the SUCRA ranking, PPV-IVR was associated with the lowest incidence of iatrogenic retinal breaks (SUCRA = 83.9%), followed by PPV-IVC (70%) and PPV-IVB (43.2%) (see **Figure 4D**).

#### 3.3.6 Reoperation rate

**Figure 5:**
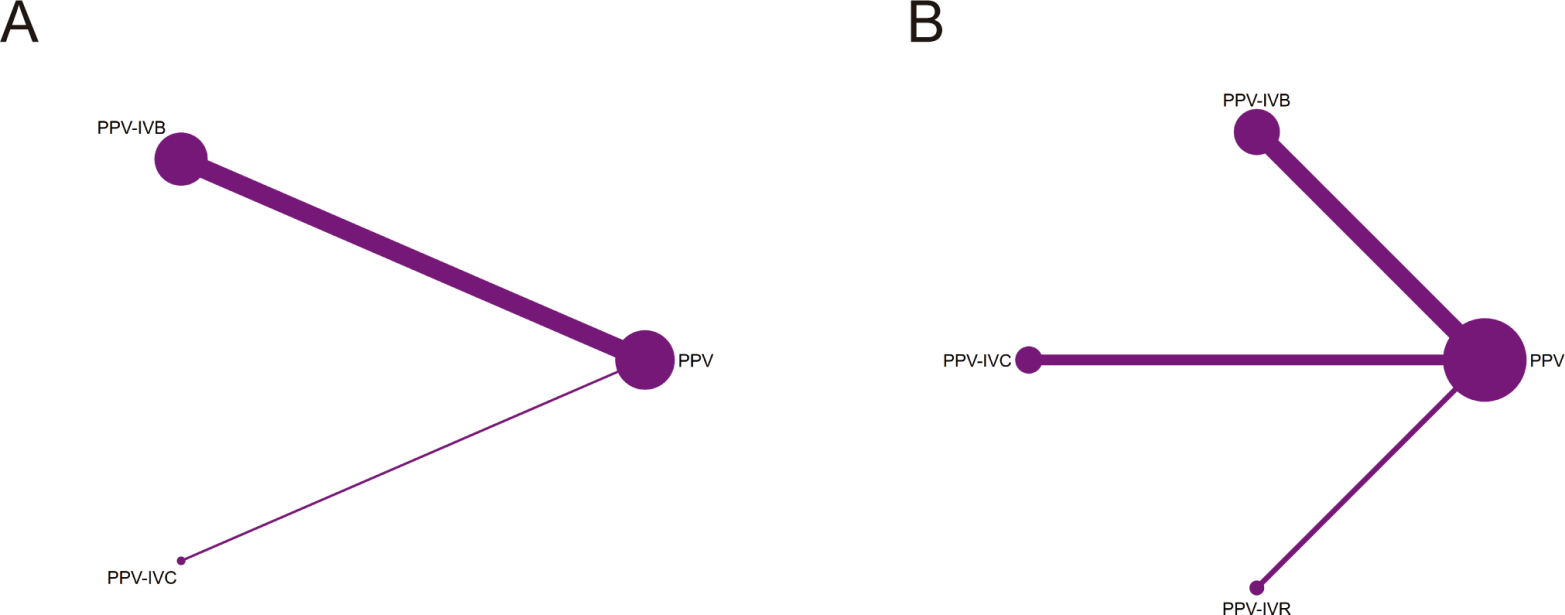
Network diagram comparing PPV with or without intravitreal anti-VEGF injection in patients with PDR. **(A)** Reoperation; **(B)** Duration of surgery.

**Figure 6:**
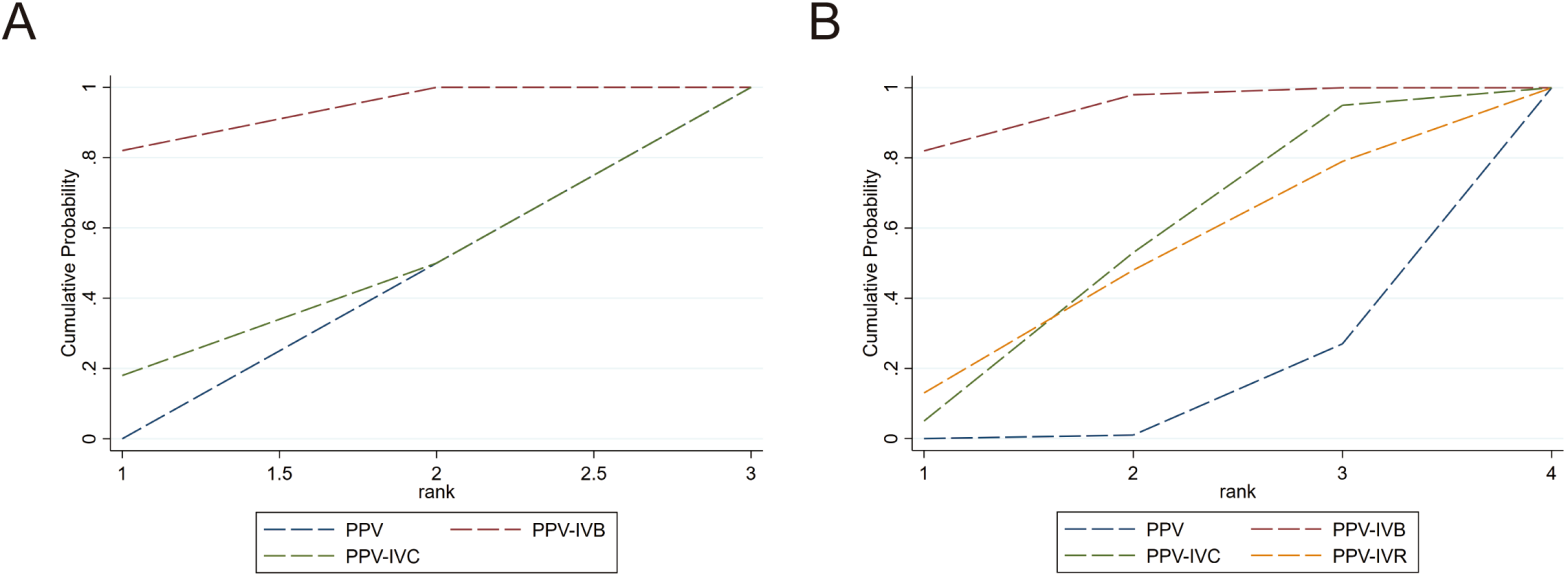
SUCRA ranking curves for PPV-related outcomes in PDR patients across different interventions. **(A)** Reoperation; **(B)** Duration of surgery.

Nine studies were included in the analysis of the reoperation rate, evaluating three intervention regimens (see **Figure 5A**). When compared to PPV alone, PPV-IVB (OR = 0.36, 95% CI: 0.21–0.63) significantly reduced the reoperation rate. PPV-IVC (OR = 1.00, 95% CI: 0.12–8.21) also reduced the reoperation rate, though this difference was not statistically significant (see **Figure 3C**). According to the SUCRA ranking, PPV-IVB was associated with the lowest reoperation rate (SUCRA = 90.8%), followed by PPV-IVC (34%) (see **Figure 6A**).

#### 3.3.7 Duration of surgery

Thirteen studies were included in the analysis of the duration of surgery, evaluating four intervention regimens (see **Figure 5B**). When compared to PPV alone, PPV-IVB (MD = -19.96, 95% CI: -29.09 to -10.82) significantly reduced the duration of surgery. PPV-IVC (MD = -8.53, 95% CI: -19.67 to -2.60) and PPV-IVR (MD = -7.62, 95% CI: -26.76 to 11.52) also reduced the duration of surgery, though these differences were not statistically significant (see **Figure 3B**). According to the SUCRA ranking, PPV-IVB was associated with the shortest duration of surgery (SUCRA = 93.5%), followed by PPV-IVC (50.7%) and PPV-IVR (46.5%) (see **Figure 6B**).

### 3.4 Subgroup analysis based on injection timing

To investigate whether the timing of preoperative anti-VEGF administration influences the relative efficacy and safety of different agents, we performed pre-specified subgroup analyses stratifying studies into two categories: the common window (5–7 days), which represents the most frequently used timing in clinical practice, and other windows (<5 days or >7 days), comprising studies with more variable intervals. The corresponding league plots are presented in **Supplementary Figure 8** and **9**.

#### 3.4.1 Common window (5–7 days)

In the sensitivity analysis restricted to studies with injection timing within the 5–7 day window, network meta-analyses were feasible for most outcomes. The results are presented below.

Intraoperative Bleeding: Four studies contributed to this analysis within the common window. IVC demonstrated the highest efficacy in reducing intraoperative bleeding (OR = 0.04, 95% CI: 0.00–0.48; SUCRA = 90.4%), followed by IVB (OR = 0.14, 95% CI: 0.03–0.59; SUCRA = 59.0%). Both anti-VEGF agents significantly outperformed PPV alone (SUCRA = 0.6%). IVR was not represented in this analysis due to the absence of studies reporting intraoperative bleeding within the 5–7 day window.

Postoperative Vitreous Hemorrhage: Six studies reported this outcome within the common window. IVB was the most effective agent (OR = 0.12, 95% CI: 0.05–0.30; SUCRA = 78.8%), followed by IVC (OR = 0.19, 95% CI: 0.07–0.54; SUCRA = 59.1%) and IVR (OR = 0.21, 95% CI: 0.01–5.07; SUCRA = 56.3%). All anti-VEGF agents showed superior efficacy compared to PPV alone (SUCRA = 5.8%). Notably, IVA was not represented in this analysis, as the only study evaluating IVA (Qu et al., 2023) had an injection timing outside the 5–7 day window.

Changes in BCVA: Four studies contributed to this analysis. IVB showed the greatest visual improvement (MD = -0.68, 95% CI: -1.29 to -0.07; SUCRA = 76.3%), closely followed by IVC (MD = -0.72, 95% CI: -1.66 to 0.22; SUCRA = 74.9%). IVR showed a more modest improvement (MD = -0.20, 95% CI: -1.03 to 0.63; SUCRA = 35.2%). All agents trended toward better visual outcomes than PPV alone (SUCRA = 13.5%).

Iatrogenic Retinal Breaks: Six studies contributed to this analysis. IVR was the most effective agent (OR = 0.12, 95% CI: 0.01–2.45; SUCRA = 72.6%), followed by IVC (OR = 0.12, 95% CI: 0.01–2.50; SUCRA = 70.9%) and IVB (OR = 0.33, 95% CI: 0.12–0.88; SUCRA = 50.7%). All anti-VEGF agents reduced the risk compared to PPV alone (SUCRA = 5.8%).

Duration of Surgery: Five studies contributed to this analysis. IVB was the most effective agent for shortening surgical duration (MD = -23.45, 95% CI: -37.15 to -9.75; SUCRA = 98.5%), followed by IVC (MD = -4.26, 95% CI: -18.16 to 9.64; SUCRA = 52.5%) and IVR (MD = 12.00, 95% CI: -17.53 to 41.53; SUCRA = 13.7%). Only IVB showed a statistically significant reduction compared to PPV alone.

The detailed network comparisons for the common window are presented in **Supplementary Figure 8**.

#### 3.4.2 Other windows (<5 days or >7 days)

Exploratory analyses of studies with injection timing outside the 5–7 day window showed directional consistency with the primary results, although confidence intervals were wider due to the heterogeneity of intervals and smaller sample sizes within specific timing subgroups.

Postoperative Vitreous Hemorrhage: Six studies contributed to this analysis. IVA demonstrated the highest efficacy (OR = 0.19, 95% CI: 0.04–0.82; SUCRA = 75.0%), followed by IVC (OR = 0.21, 95% CI: 0.05–0.82; SUCRA = 70.3%) and IVB (OR = 0.32, 95% CI: 0.09–1.23; SUCRA = 52.2%). All agents were superior to PPV alone (SUCRA = 2.5%).

Changes in BCVA: Four studies contributed to this analysis. IVR showed the greatest visual improvement (MD = -0.24, 95% CI: -0.72 to 0.24; SUCRA = 72.9%), followed by IVB (MD = -0.15, 95% CI: -0.44 to 0.13; SUCRA = 62.2%). Both agents outperformed PPV alone (SUCRA = 15.0%).

Iatrogenic Retinal Breaks: Four studies contributed to this analysis. IVR remained the most effective agent (OR = 0.07, 95% CI: 0.01–0.71; SUCRA = 95.5%), significantly outperforming IVB (OR = 0.42, 95% CI: 0.20–0.86; SUCRA = 53.4%) and PPV alone (SUCRA = 1.0%).

Duration of Surgery: Five studies contributed to this analysis. IVB and IVR showed comparable efficacy in reducing surgical time. IVB reduced surgical time by 18.67 minutes (95% CI: -33.43 to -3.91; SUCRA = 74.0%), while IVR showed a reduction of 19.10 minutes (95% CI: -44.16 to 5.96; SUCRA = 72.2%). Both agents outperformed PPV alone (SUCRA = 3.8%).

The detailed network comparisons for the other windows are presented in **Supplementary Figure 9**.

#### 3.4.3 Summary of subgroup findings

These subgroup analyses demonstrate that the relative advantages of different anti-VEGF agents are generally consistent across timing windows, though some variations exist due to differences in the distribution of studies and agents across windows. Key findings include:

Intraoperative bleeding (common window): IVC ranked highest, while IVR was not represented.

Postoperative vitreous hemorrhage: IVB ranked highest in the common window, while IVA ranked highest in other windows (where it was exclusively represented).

Visual improvement: IVB and IVC showed comparable benefits in the common window; IVR led in other windows.

Iatrogenic retinal breaks: IVR consistently ranked highest across both windows.

Surgical duration: IVB consistently demonstrated the greatest reduction across both windows.

The variations observed across windows primarily reflect the different compositions of studies and agents available for analysis, rather than genuine inconsistencies in treatment effects. Notably, the rankings within each window were internally consistent with the data available, and the overall pattern of agent-specific advantages, such as IVR for retinal breaks and IVB for surgical efficiency, remained robust when sufficient data were available for comparison.

The consistency of results between the common window and the primary analysis confirms the robustness of our main conclusions, while the exploratory analyses of other windows provide supportive evidence and highlight potential differential effects that warrant further investigation.

### 3.5 Sensitivity analysis, meta-regression, and publication bias

To assess the influence of individual studies on the network effect estimates, sensitivity analysis was conducted for all outcome measures using the leave-one-out method. In each iteration, one study was sequentially excluded, and the remaining studies were reanalyzed through a consistency random-effects network meta-analysis. The results demonstrated that, regardless of the study excluded, the direction of the combined effect for each anti-VEGF agent in combination with PPV compared to PPV alone remained consistent. The changes in effect size were marginal, the confidence intervals were largely overlapping, and there was no significant alteration in statistical significance, suggesting that the findings were robust (**Supplementary 3**).

For the six outcome measures, univariate network meta-regression analyses were subsequently performed, incorporating disease severity (defined by the presence of tractional retinal detachment) and age as covariates. This was done to explore potential influences of these factors on the relative effects of different anti-VEGF treatments in comparison to standard therapy. The regression analysis revealed that neither disease severity nor age had a significant impact on the effect sizes of any intervention relative to standard treatment (**Supplementary 4**).

Additionally, we performed network meta-regression using injection-to-surgery interval as a continuous covariate (**Supplementary Table 4**.13–4.18). The results showed no significant modification of treatment effects by injection timing for intraoperative bleeding (coefficient: -0.33; 95% CI: -1.23 to 0.56; p = 0.46), postoperative vitreous hemorrhage (coefficient: -0.06; 95% CI: -0.36 to 0.25; p = 0.71), changes in BCVA (coefficient: -0.12; 95% CI: -0.32 to 0.08; p = 0.25), iatrogenic retinal breaks (coefficient: -0.16; 95% CI: -0.42 to 0.10; p = 0.23), reoperation (coefficient: 0.04; 95% CI: -0.31 to 0.40; p = 0.82), or duration of surgery (coefficient: -0.57; 95% CI: -4.78 to 3.64; p = 0.79). These findings suggest that, within the observed range of 1 to 20 days, the timing of preoperative anti-VEGF administration did not significantly alter the relative efficacy of the agents.

Moreover, funnel plots were generated for all outcome measures to evaluate publication bias. The distribution of study points was generally symmetrical, with no evident outliers, indicating a minimal risk of publication bias in this study (**Supplementary Figures 2-7**).

### 3.6 GRADE assessment

The evidence quality for all outcome measures was evaluated using the CINeMA framework, revealing variability in evidence levels, ranging from *high* to *very low*. Regarding the comparison of the outcome *intraoperative bleeding*, 5 out of 6 comparisons (83%) were rated as low, while 1 comparison (17%) was rated as very low. For the outcome *postoperative vitreous hemorrhage*, 3 out of 10 comparisons (30%) were rated as moderate, and 7 comparisons (70%) were rated as low. In terms of *changes in BCVA*, 1 out of 6 comparisons (17%) was rated as moderate, and 5 comparisons (83%) were rated as low. For *iatrogenic retinal breaks*, 2 out of 6 comparisons (33%) were rated as moderate, while 4 comparisons (67%) were rated as low. Concerning *reoperation*, 1 out of 3 comparisons (33%) was rated as moderate, and 2 comparisons (67%) were rated as low. Lastly, for the outcome *duration of surgery*, 5 out of 6 comparisons (83%) were rated as low, and 1 comparison (17%) was rated as very low (**Supplementary 5**).

## 4 Discussion

To the best of our knowledge, this study represents the first systematic assessment of the efficacy and safety of various anti-VEGF agents — bevacizumab, ranibizumab, conbercept, and aflibercept — combined with PPV for the treatment of PDR using a NMA approach. By comprehensively evaluating outcomes such as intraoperative bleeding, postoperative vitreous rebleeding, visual function recovery (BCVA changes), as well as intraoperative iatrogenic retinal breaks, reoperation rates, and surgery duration, this study elucidates the *advantage profiles* of different anti-VEGF regimens across multiple clinical objectives. These findings provide robust, quantifiable evidence to guide the tailored selection of adjunctive perioperative medications. The key findings are as follows:

(1) Compared to PPV alone, all anti-VEGF agents in combination with surgery significantly reduced the risk of intraoperative bleeding, with ranibizumab demonstrating the most favorable performance.
(2) In preventing postoperative vitreous rebleeding, aflibercept, conbercept, and bevacizumab showed substantial superiority over surgery alone, with aflibercept leading the efficacy ranking.
(3) Regarding postoperative visual acuity improvement, all drugs exhibited a positive trend, with conbercept showing the highest degree of improvement.
(4) In terms of reducing iatrogenic retinal breaks, ranibizumab and bevacizumab significantly outperformed surgery alone, with ranibizumab achieving the highest benefit.
(5) For reducing reoperation rates and shortening surgery duration, bevacizumab demonstrated notable advantages, consistently delivering benefits across other safety and efficacy indicators.This makes bevacizumab a balanced option for ensuring intraoperative safety, anatomical success, and surgical efficiency.

The differences observed between various anti-VEGF agents across different outcomes may be attributed to their molecular structures, tissue distribution, and mechanisms of action. Bevacizumab, a full-length humanized monoclonal antibody, has a relatively large molecular weight and a prolonged half-life in the vitreous, potentially facilitating more sustained VEGF inhibition (39). This prolonged action may promote the regression of neovascularization, stabilize lesions, and reduce the risk of re-intervention due to persistent active foci. Furthermore, it may contribute to a shorter surgical duration by mitigating intraoperative challenges, which correlates with its relative advantages in reoperation rates and surgical duration. In contrast, ranibizumab, as an antibody fragment, likely exhibits superior tissue penetration, enabling more rapid VEGF suppression and potentially aiding in the immediate reduction of neovascular leakage during surgery (40). This mechanism may account for its enhanced performance in minimizing intraoperative complications, such as bleeding and iatrogenic retinal breaks. Aflibercept, a VEGF receptor fusion protein, demonstrates robust and high-affinity binding to VEGF-A, VEGF-B, and PlGF, which may result in more comprehensive and potent inhibition of the VEGF pathway (41). This broad action likely underpins its advantages in preventing long-term postoperative vitreous hemorrhage. Conbercept, a multi-target fusion protein, shows a promising trend in improving visual acuity, likely attributable to its unique molecular structure and broader inhibition of the VEGF family (42). It is important to acknowledge that while these mechanistic explanations are biologically plausible and consistent with clinical outcomes, further direct comparative and mechanistic studies are necessary for validation.

The findings of this study align with the conclusions of several recent systematic reviews. The most recent meta-analysis by Fan et al. (16) demonstrated that preoperative anti-VEGF therapy significantly reduced the incidence of intraoperative vitreous hemorrhage (RR = 0.38) and iatrogenic retinal breaks (RR = 0.56), shortened the duration of surgery (WMD = -24.04 minutes), and improved postoperative best-corrected visual acuity (WMD = -0.18). These results strongly support the advantages of anti-VEGF therapy over surgery alone, as observed in the present study. Importantly, the timing of drug administration is a critical factor influencing efficacy. Wang et al. (43) conducted a network meta-analysis specifically examining the timing of conbercept injection. Their findings indicated that administering the drug 3–7 days prior to surgery (medium to long intervals) significantly improved both intraoperative and postoperative outcomes, whereas immediate or intraoperative administration did not provide additional benefits. These findings suggest that optimizing the administration window (e.g., 5–7 days before surgery) could further enhance the adjunctive benefits of anti-VEGF therapy in clinical practice. Our network meta-regression extended this line of inquiry by examining whether the relative efficacy of different anti-VEGF agents varies across the broader 1–20 day range of injection intervals observed in the included studies. Notably, we found that the injection-to-surgery interval did not significantly modify the treatment effects on any of the primary or secondary outcomes (**Supplementary Table 4**.13-4.18). This finding suggests that while an optimal window of 5–7 days may maximize the overall benefits of anti-VEGF pretreatment, the comparative advantages of specific agents remain robust across the clinically relevant range of injection timing. Specifically, IVR demonstrated consistent benefits for intraoperative hemostasis, IVA for preventing postoperative vitreous hemorrhage, and IVB for shortening surgical duration. This was further supported by our subgroup analyses, which showed that these agent-specific advantages were generally consistent across different timing windows, despite some variations attributable to the distribution of available studies (**Supplementary Figures 8** and **9**). The apparent lack of a dose-response relationship may be due to the concentration of studies within the narrow 5–7 day window, limiting statistical power to detect a linear effect across the full range. Nevertheless, these results reinforce the robustness of our primary conclusions and suggest that clinicians can confidently select anti-VEGF agents based on their distinct advantage profiles, regardless of whether surgery is scheduled at the optimal 5–7 day window or at alternative intervals within the 1–20 day range. Additionally, with the growing trend of minimally invasive surgery, Pei et al. (13) confirmed in their meta-analysis that preoperative anti-VEGF therapy effectively reduces intraoperative complications and early postoperative hemorrhage in small-gauge vitrectomy (23G/25G/27G). This evidence suggests that the conclusions of this study are applicable across different surgical techniques, with the combination of anti-VEGF pretreatment and minimally invasive surgery enhancing the overall safety of PDR surgery.

Based on this body of evidence, clinical decision-making should prioritize treatment goals when selecting an anti-VEGF agent. For those aiming to shorten the duration of surgery, reduce reoperation rates, and balance cost-effectiveness, bevacizumab is a comprehensive choice. In cases with a particularly high risk of intraoperative bleeding, ranibizumab offers superior control over immediate hemorrhage. If the primary concern is postoperative vitreous rebleeding, aflibercept may be the optimal option. Finally, if visual recovery is the key priority and preoperative administration can be planned several days in advance, conbercept shows promising potential. This study provides the first direct comparison and ranking of these four major anti-VEGF agents, offering valuable evidence for their differentiated use through network meta-analysis.

The strengths of this study are as follows: 1) Innovative Study Design: This is the first network meta-analysis comparing four major anti-VEGF agents in combination with PPV for the treatment of PDR, addressing a critical gap in direct comparative evidence in this field. 2) Methodological Rigor: The study adhered to the PRISMA-NMA guidelines, incorporating a pre-registered protocol, a consistency model, and comprehensive sensitivity analyses, meta-regression, and GRADE evidence grading, ensuring the robustness and reliability of the findings. 3) Comprehensive Outcome Measures: A broad array of outcomes were evaluated, including surgical safety, anatomical success, and functional recovery, providing a thorough assessment of both efficacy and safety. 4) Systematic Quality Assessment: The application of the RoB 2 tool for a stringent risk-of-bias assessment of the included studies resulted in an overall acceptable quality, thus enhancing the credibility of the conclusions.

Despite the valuable insights derived from this study, several limitations should be considered. First, certain comparisons, particularly those involving aflibercept and conbercept, were based on relatively small sample sizes due to the scarcity of direct evidence, which may affect the precision of estimates and statistical power. Second, the studies included were predominantly conducted in specific regions, which may limit the generalizability of the results to diverse populations and healthcare settings. Third, there were variations in study design, including differences in follow-up duration, injection-to-surgery intervals, surgeon experience, and surgical techniques, as well as minor discrepancies in the definitions of key outcomes such as intraoperative bleeding and postoperative vitreous hemorrhage (**Supplementary Table 3**), although sensitivity analyses excluding studies with non-standard definitions did not materially alter the results, suggesting that our findings are robust to these variations, which may introduce heterogeneity. Lastly, most outcome networks lacked closed loops, with drug comparisons primarily relying on indirect evidence. Consequently, we were unable to perform global or local inconsistency tests, and the transitivity assumption could not be fully verified empirically. Although we downgraded the indirectness dimension in the GRADE assessment and interpreted the findings cautiously, the strength of this evidence remains weaker than that of direct comparisons from large-scale, head-to-head RCTs. This limitation should be considered when applying our findings to clinical practice. For some outcomes, such as changes in BCVA, which did not reach statistical significance, further high-quality studies are needed to confirm these findings.

Future studies should focus on large-scale, multicenter, head-to-head randomized controlled trials that directly compare the efficacy and safety of different anti-VEGF agents during the perioperative period for PDR. These studies should also include stratified or predefined subgroup analyses to assess key effect modifiers (such as baseline bleeding risk, degree of fibrosis, injection-to-surgery interval, minimally invasive gauge size, and injection technique). Additionally, integrating potential biomarkers (such as VEGF levels, inflammatory markers, or fibrosis-related indicators) could help explore personalized treatment strategies. Furthermore, it is essential to determine the optimal administration window and dosing regimen for different drugs in the context of minimally invasive surgery, in order to develop more refined and broadly applicable clinical guidelines.

## 5 Conclusion

This network meta-analysis offers a comprehensive comparison of the efficacy and safety of four major anti-VEGF agents (bevacizumab, ranibizumab, conbercept, and aflibercept), when used in combination with pars plana vitrectomy (PPV) for treating proliferative diabetic retinopathy (PDR). Our findings reveal distinct advantages of each agent in the perioperative management of PDR. Ranibizumab demonstrated superior efficacy in reducing intraoperative bleeding, while aflibercept was most effective in preventing postoperative vitreous hemorrhage. Conbercept showed promising results for improving postoperative vision, and bevacizumab was the most efficient in enhancing surgical efficiency and reducing reoperation rates. These results highlight the importance of personalized treatment decisions based on the specific clinical goals of PDR management. For optimal surgical outcomes, clinicians should select anti-VEGF agents based on factors such as the risk of intraoperative bleeding, the need for visual recovery, and the anticipated surgical complexity. While bevacizumab offers a balanced approach for surgical efficiency and safety, other agents may be more suitable for specific complications, such as vitreous hemorrhage or retinal breaks. This study provides valuable evidence to inform the selection of adjunctive anti-VEGF therapy in clinical practice, and further head-to-head trials are needed to confirm these findings and refine clinical guidelines.

## Data Availability

The original contributions presented in the study are included in the article/**Supplementary Material**. Further inquiries can be directed to the corresponding author.
