# Supplementary material for: Efficacy and safety of different anti-VEGF agents combined with pars plana vitrectomy in proliferative diabetic retinopathy: a systematic review and network meta-analysis of randomized controlled trials
Source: Front Endocrinol (Lausanne). 2026 Mar 25;17:1772351. doi: 10.3389/fendo.2026.1772351 (PMC13056647; doi:10.3389/fendo.2026.1772351)
Supplement: Supplementary file 1 [file Supplementaryfile1.pdf]

Supplementary Material

1 Supplementary Figures and Tables

1.1 Supplementary Table

1.2 Supplementary Table 1. PRISMA NMA Checklist of Items to Include When Reporting a Systematic Review Involving a Network Meta-analysis

Section/Topic	Item	Checklist Item	Reported on Page
TITLE			
Title	1	Identify the report as a systematic review *incorporating* a network meta-analysis (or related form of meta-analysis).	1
ABSTRACT			
Structured summary	2	Provide a structured summary including, as applicable:<br>**Objective:** main objectives<br>**Methods:** data sources; study eligibility criteria, participants, and interventions; study appraisal; and *synthesis methods, such as network meta-analysis.*<br>**Results:** number of studies and participants identified; summary estimates with corresponding confidence/credible intervals; treatment rankings may also be discussed. Authors may choose to summarize pairwise comparisons against a chosen treatment included in their analyses for brevity.<br>**Discussion/Conclusions:** limitations; conclusions and implications of findings.<br>**Other:** systematic review registration number with registry name.	1-2

INTRODUCTION			
Rationale	3	Describe the rationale for the review in the context of what is already known*, including mention of why a network meta-analysis has been conducted.*	2
Objectives	4	Provide an explicit statement of questions being addressed, with reference to participants, interventions, comparisons, outcomes, and study design (PICOS).	2
METHODS			
Protocol and registration	5	Indicate whether a review protocol exists and if and where it can be accessed (e.g., Web address); and, if available, provide registration information, including registration number.	3
Eligibility criteria	6	Specify study characteristics (e.g., PICOS, length of follow-up) and report characteristics (e.g., years considered, language, publication status) used as criteria for eligibility, giving rationale. *Clearly describe eligible treatments included in the treatment network, and note whether any have been clustered or merged into the same node (with justification).*	3-4
Information sources	7	Describe all information sources (e.g., databases with dates of coverage, contact with study authors to identify additional studies) in the search and date last searched.	4
Search	8	Present full electronic search strategy for at least one database, including any limits used, such that it could be repeated.	3
Study selection	9	State the process for selecting studies (i.e., screening, eligibility, included in systematic review, and, if applicable, included in the meta-analysis).	3-4
Data collection process	10	Describe method of data extraction from reports (e.g., piloted forms, independently, in duplicate) and any processes for obtaining and confirming data from	4

		investigators.	
Data items	11	List and define all variables for which data were sought (e.g., PICOS, funding sources) and any assumptions and simplifications made.	3-4
Geometry of the network	S1	Describe methods used to explore the geometry of the treatment network under study and potential biases related to it. This should include how the evidence base has been graphically summarized for presentation, and what characteristics were compiled and used to describe the evidence base to readers.	4-5
Risk of bias within individual studies	12	Describe methods used for assessing risk of bias of individual studies (including specification of whether this was done at the study or outcome level), and how this information is to be used in any data synthesis.	5-6
Summary measures	13	State the principal summary measures (e.g., risk ratio, difference in means). Also describe the use of additional summary measures assessed, such as treatment rankings and surface under the cumulative ranking curve (SUCRA) values, as well as modified approaches used to present summary findings from meta-analyses.	4-6
Planned methods of analysis	14	Describe the methods of handling data and combining results of studies for each network meta-analysis. This should include, but not be limited to:<br>• Handling of multi-arm trials;<br>• Selection of variance structure;<br>• Selection of prior distributions in Bayesian analyses; and<br>• Assessment of model fit.	4-6
Assessment of Inconsistency	S2	Describe the statistical methods used to evaluate the agreement of direct and indirect evidence in the treatment network(s) studied. Describe efforts taken to address its presence when found.	4-5
Risk of bias across	15	Specify any assessment of risk of bias that may affect the cumulative evidence (e.g., publication bias,	4-5

studies		selective reporting within studies).	
Additional analyses	16	Describe methods of additional analyses if done, indicating which were pre-specified. This may include, but not be limited to, the following:<br>• Sensitivity or subgroup analyses;<br>• Meta-regression analyses;<br>• Alternative formulations of the treatment network; and<br>• Use of alternative prior distributions for Bayesian analyses (if applicable).	4-5
RESULTS			
Study selection	17	Give numbers of studies screened, assessed for eligibility, and included in the review, with reasons for exclusions at each stage, ideally with a flow diagram.	6-7
Presentation of network structure	S3	Provide a network graph of the included studies to enable visualization of the geometry of the treatment network.	6-12
Summary of network geometry	S4	Provide a brief overview of characteristics of the treatment network. This may include commentary on the abundance of trials and randomized patients for the different interventions and pairwise comparisons in the network, gaps of evidence in the treatment network, and potential biases reflected by the network structure.	6-12
Study characteristics	18	For each study, present characteristics for which data were extracted (e.g., study size, PICOS, follow-up period) and provide the citations.	6-12
Risk of bias within studies	19	Present data on risk of bias of each study and, if available, any outcome level assessment.	6-12
Results of individual studies	20	For all outcomes considered (benefits or harms), present, for each study: 1) simple summary data for each intervention group, and 2) effect estimates and confidence intervals. *Modified approaches may be*	6-12

		needed to deal with information from larger networks.	
Synthesis of results	21	Present results of each meta-analysis done, including confidence/credible intervals. In larger networks, authors may focus on comparisons versus a particular comparator (e.g. placebo or standard care), with full findings presented in an appendix. League tables and forest plots may be considered to summarize pairwise comparisons. If additional summary measures were explored (such as treatment rankings), these should also be presented.	6-12
Exploration for inconsistency	S5	Describe results from investigations of inconsistency. This may include such information as measures of model fit to compare consistency and inconsistency models, *P* values from statistical tests, or summary of inconsistency estimates from different parts of the treatment network.	6-12
Risk of bias across studies	22	Present results of any assessment of risk of bias across studies for the evidence base being studied.	6-12
Results of additional analyses	23	Give results of additional analyses, if done (e.g., sensitivity or subgroup analyses, meta-regression analyses*, alternative network geometries studied, alternative choice of prior distributions for Bayesian analyses,* and so forth).	6-12
DISCUSSION			
Summary of evidence	24	Summarize the main findings, including the strength of evidence for each main outcome; consider their relevance to key groups (e.g., healthcare providers, users, and policy-makers).	12-15
Limitations	25	Discuss limitations at study and outcome level (e.g., risk of bias), and at review level (e.g., incomplete retrieval of identified research, reporting bias). *Comment on the validity of the assumptions, such as transitivity and consistency. Comment on any concerns regarding network geometry (e.g., avoidance of certain*	12-15

		comparisons).	
Conclusions	26	Provide a general interpretation of the results in the context of other evidence, and implications for future research.	15
FUNDING			
Funding	27	Describe sources of funding for the systematic review and other support (e.g., supply of data); role of funders for the systematic review. This should also include information regarding whether funding has been received from manufacturers of treatments in the network and/or whether some of the authors are content experts with professional conflicts of interest that could affect use of treatments in the network.	16

1.3 **Supplementary Table 2.** Literature Search Strategy

Supplementary Table 2. Literature Search Strategy	
Pubmed	((((("Diabetic Retinopathy"[Mesh]) OR (Diabetic Retinopathies[Title/Abstract])) OR (Retinopathies, Diabetic[Title/Abstract])) OR (Retinopathy, Diabetic[Title/Abstract])) OR (Proliferative Diabetic Retinopathy[Title/Abstract])) AND ((((((((Bevacizumab[Title/Abstract]) OR (Ranibizumab[Title/Abstract])) OR (Conbercept[Title/Abstract])) OR (aflibercept[Title/Abstract])) OR (anti-vascular endothelial growth factor[Title/Abstract]) OR (pars plana vitrectomy[Title/Abstract]))) OR (microincision vitrectomy surgery[Title/Abstract])) AND (randomized controll edtrial[Filter])
Web of	1: TS=("diabetic retinopath*" OR "proliferative diabetic retinopath*")

Science	2: TS=(bevacizumab OR ranibizumab OR conbercept OR aflibercept OR "anti vascular endothelial growth factor" OR "anti vegf" OR "anti-vegf" OR "pars plana vitrectom*" OR "microincision vitrectomy surger*")<br>3: TS=(random* OR trial OR placebo) OR TI=(randomized OR randomised)<br><br>4: #3 AND #2 AND #1
Cochrane	#1 "diabetic retinopathy" OR "Diabetic Retinopathies" OR "Retinopathies, Diabetic" OR "Retinopathy, Diabetic" OR "proliferative diabetic retinopathy"<br>#2 bevacizumab OR ranibizumab OR conbercept OR aflibercept OR "anti vascular endothelial growth factor" OR "anti vegf" OR "anti-vegf"<br>#3 "pars plana vitrectom" OR "microincision vitrectomy surger"<br>#4 #2 OR #3<br>#5 #1 AND #4
Embase	#9. #3 AND #7 AND #8<br>#8. 'randomized controlled trial'/de OR 'randomized controlled trial':ti,ab,kw OR 'randomised controlled trial':ti,ab,kw OR 'rct':ti,ab,kw<br>#7. #4 OR #5 OR #6<br>#6. 'pars plana vitrectom*':ti,ab,kw OR 'microincision vitrectomy surger*':ti,ab,kw<br>#5. 'pars plana vitrectomy'/exp<br>#4. 'bevacizumab':ti,ab,kw OR 'ranibizumab':ti,ab,kw OR 'conbercept':ti,ab,kw OR 'aflibercept':ti,ab,kw OR 'anti vascular endothelial growth factor':ti,ab,kw OR 'anti vegf':ti,ab,kw<br>#3. #1 OR #2<br>#2. 'diabetic retinopath*':ti,ab,kw OR 'proliferative diabetic retinopath*':ti,ab,kw<br>#1. 'diabetic retinopathy'/exp

1.4 **Supplementary Table 3.** Definitions of Outcome Measures Across Included Studies

No.	First Author & Publication Year	Intraoperative Bleeding Definition	Postoperative Vitreous Hemorrhage Definition	Reoperation Definition
1	Arevalo JF 2019(10)	Grade 2 bleeding (moderate to severe bleeding requiring endodiathermy or with formation of broad sheets of clots)	Vitreous hemorrhage within the first month after surgery	Second vitrectomy for recurrent TRD or dense VH
2	El-Batarny AM 2008(21)	Frequency of bleeding attacks per case; use of endodiathermy recorded	Postoperative bleeding reported as present/absent	Subsequent surgeries for redetachment or cataract
3	Comyn O 2017(11)	Intraoperative bleeding score (0–3) based on severity	Vitreous cavity hemorrhage graded 0–3 at 6 and 12 weeks	Not reported
4	Ahn J 2011(22)	Not directly defined; intraoperative hemostasis methods recorded	Recurrent VH defined as new episode of grade ≥1 occurring >1 week postop; early ≤4 weeks, late >4 weeks	Reoperation for recurrent VH (vitreous washout)
5	Yang X 2016(12)	Severity graded 0–3 (0 none, 1 minor, 2 moderate requiring endodiathermy, 3 severe covering half of posterior pole)	Recurrent VH defined as new grade ≥1 after 1 week postop; early ≤4 weeks, late >4 weeks	Not reported
6	Su L 2016(23)	Intraoperative bleeding requiring endodiathermy or causing iatrogenic breaks	Severe recurrent VH with no fundus details at 3 months	Second surgery for recurrent VH
7	Hernández-Da MSE 2010(24)	Graded 1–3: 1 minor, 2 moderate requiring endodiathermy, 3 thick clot covering posterior pole	Postoperative bleeding recorded as persistent hemorrhage	Not explicitly defined; cataract extraction and re-PPV mentioned
8	Farahvash MS 2011(25)	Scoring system: 0 mild oozing, 1 controlled by IOP elevation, 2 controlled by endodiathermy, 3 uncontrolled	Early (≤1 month) and late (>1 month) postoperative VH	Not reported
9	Ahmadieh H 2009(26)	Not directly defined; intraoperative bleeding incidence recorded	Early postoperative VH (≤4 weeks) graded per DRVS (0–3)	Not reported
10	di Lauro R 2010(27)	Intraoperative bleeding recorded (mild/severe) and need for endodiathermy	Severe recurrent VH with no fundus details at 3 and 6 months	Further surgery with silicone oil due to VH recurrence
11	Faisal SM 2018(28)	Graded as no, mild (stopped by infusion pressure), severe (required diathermy)	Not reported	Not reported
12	Li S 2022(29)	Intraoperative bleeding presence and use of endodiathermy recorded	Postoperative VH graded per DRVS at follow-ups	Second surgery for recurrent VH (mentioned)
13	Manabe A 2015(30)	Number of endodiathermy spots used	Postoperative recurrent VH defined as bleeding obscuring optic disc; early ≤4 weeks	Reoperation due to recurrent VH within 4 weeks

14	Modarres M 2009(31)	Number of endodiathermy and backflush applications	Significant postoperative VH obscuring fundus details	Reoperation for retinal detachment or silicone oil removal
15	Rizzo S 2008(32)	Intraoperative bleeding recorded as mild (controlled by pressure) or severe (required diathermy)	Not explicitly defined	Not reported
16	Sohn EH 2012(33)	Graded 0–2: 0 none, 1 minor (spontaneous/ pressure), 2 moderate-severe (diathermy/clots)	Not reported	Not reported
17	Zaman Y 2013(34)	Not explicitly defined; bleeding frequency recorded	Early postoperative VH (≤4 weeks)	Not reported
18	Ding Y 2023(35)	Hemorrhage score 0–3 based on severity; number of electrocoagulation applications	Early (≤1 month) and late (>1 month) postoperative VH	Not reported
19	Qu JF 2023(13)	Not directly defined; intraoperative bleeding controlled by diathermy/pressure	Postoperative VH graded per DRVS at 1 week, 1,2,3 months	Not reported (reoperations excluded)
20	Yang Z 2023(36)	Intraoperative bleeding recorded as presence/absence	Postoperative bleeding within 3 days after surgery	Not reported
21	Jiang T 2020(37)	Not directly defined; intraoperative bleeding controlled by diathermy/pressure	Early (≤1 month) and late (>1 month) postoperative VH; ITVC defined as days for VH to clear	Second surgery for recurrent VH
22	Ren X 2019(38)	Not directly defined	Recurrent VH recorded at follow-ups	Not reported

Abbreviations: DRVS = Diabetic Retinopathy Vitrectomy Study grading system; ITVC = initial time of vitreous clearing; IOP = intraocular pressure.

2 Supplementary Figures

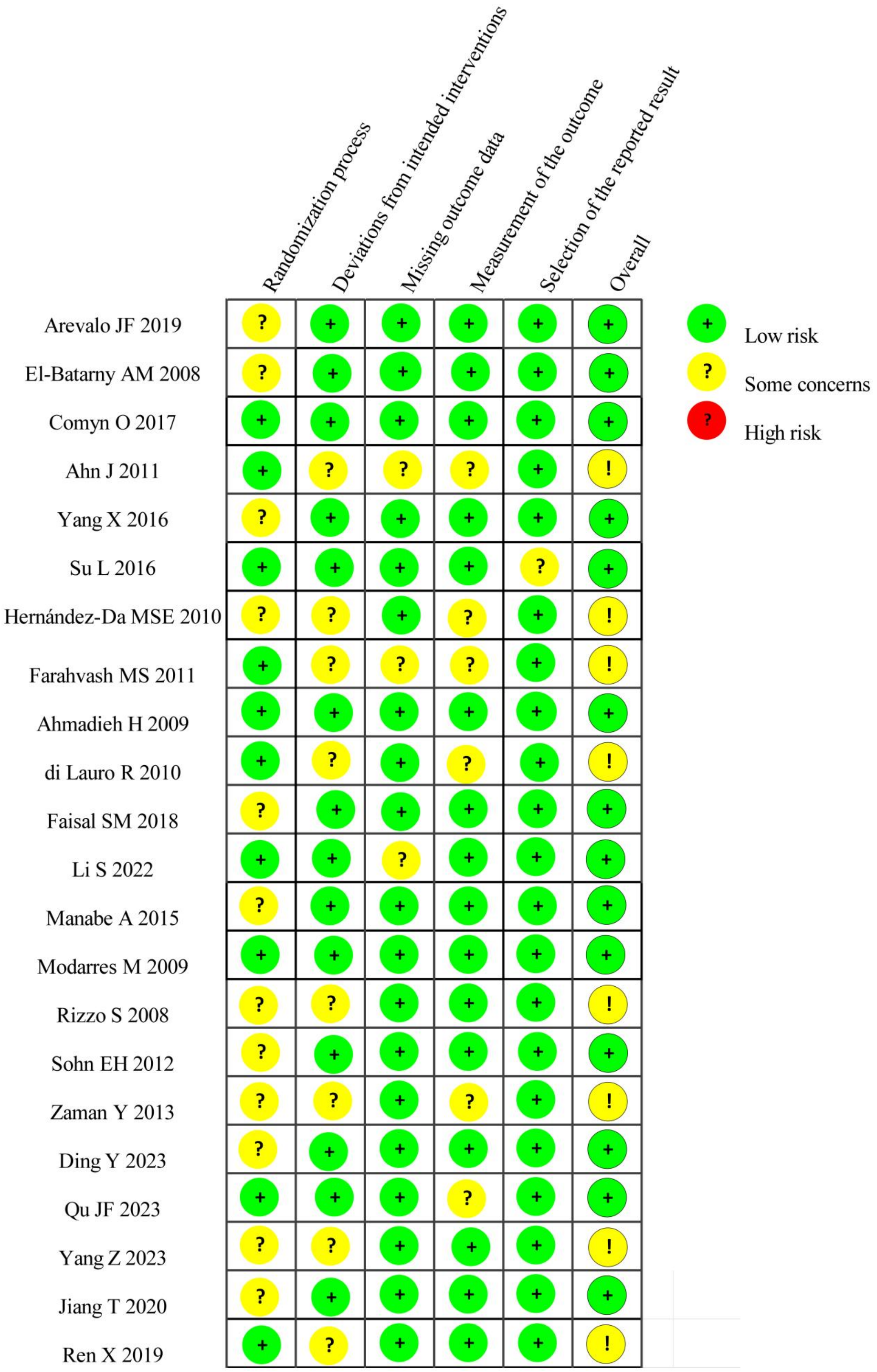

Supplementary Figure 1. Risk of Bias Assessment for Included Studies.The rows display individual studies, with the color-coded circles indicating the risk level for each domain: green circles (+)

represent low risk, yellow circles (?) indicate some concerns, and red circles (!) denote high risk. The overall assessment reflects the cumulative risk across all domains for each study.

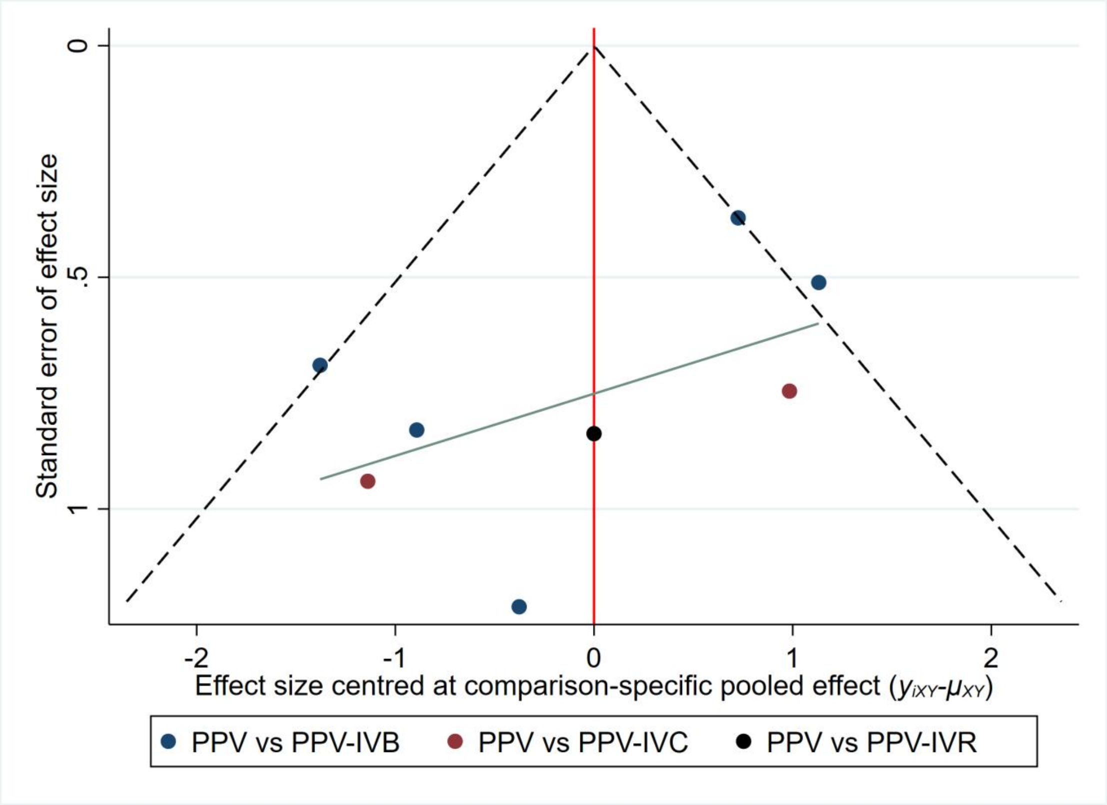

Supplementary Figure 2. The funnel plot of Intraoperative bleeding.

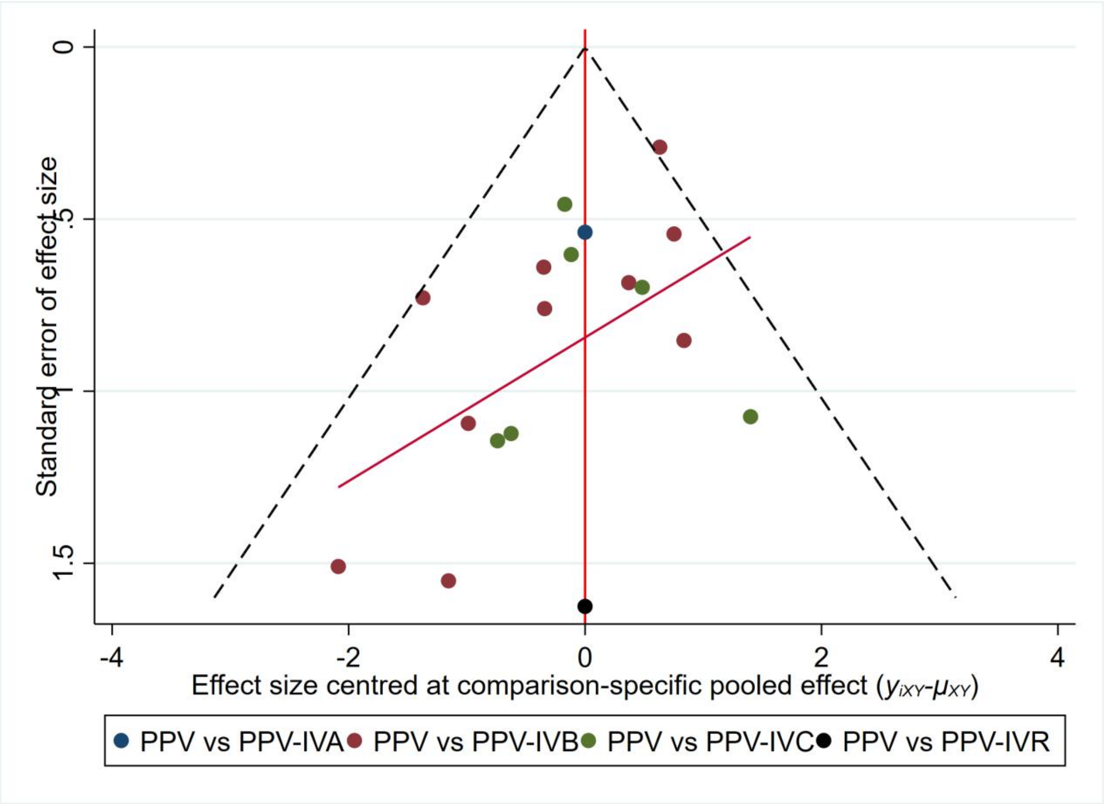

Supplementary Figure 3. The funnel plot of Postoperative vitreous hemorrhage.

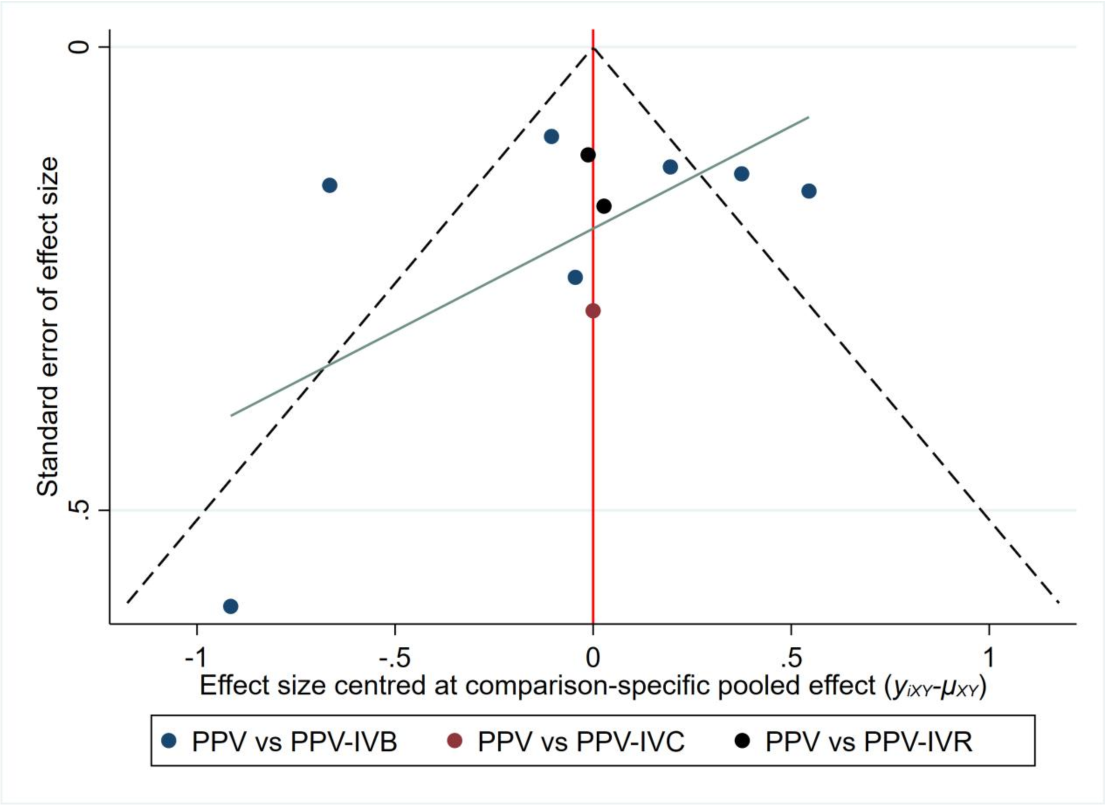

Supplementary Figure 4. The funnel plot of Changes in BCVA.

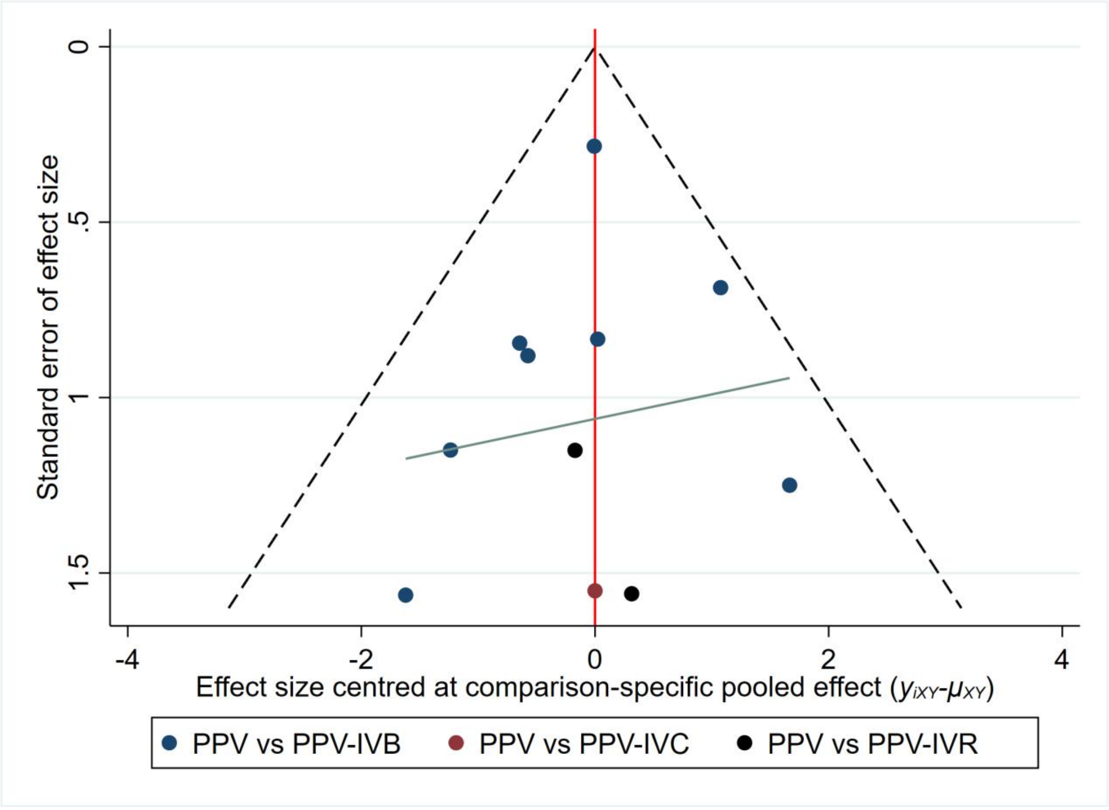

Supplementary Figure 5. The funnel plot of Iatrogenic retinal breaks.

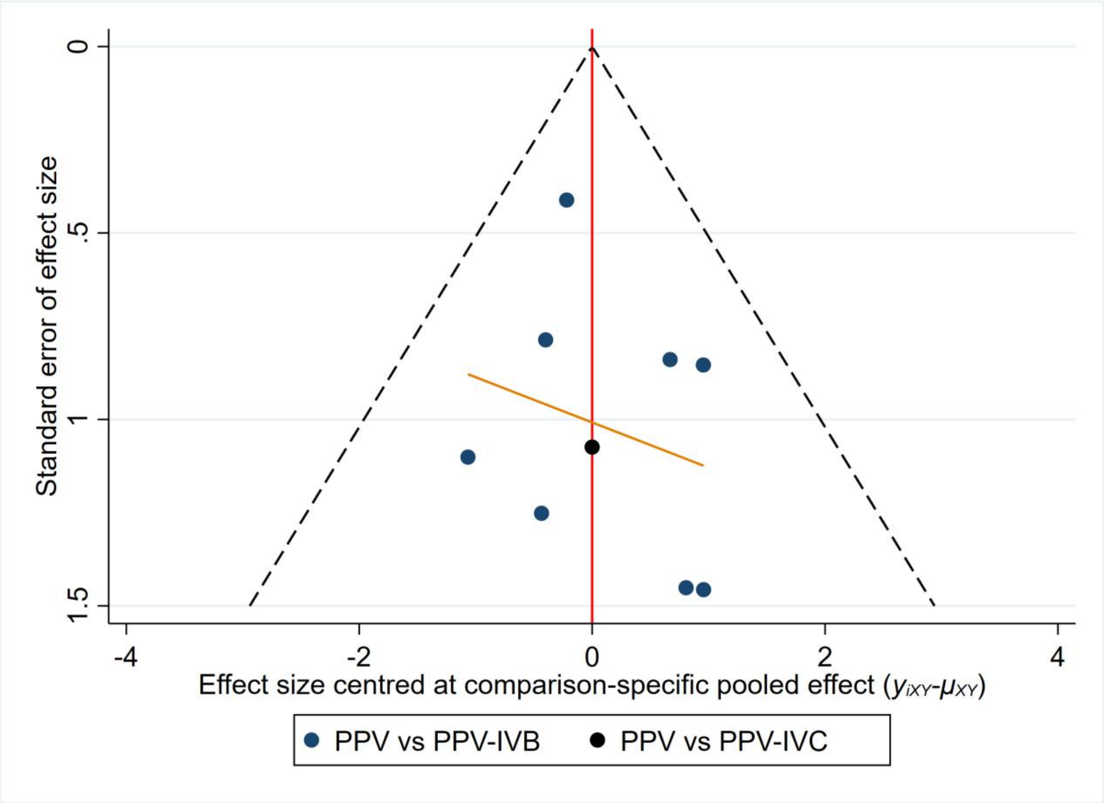

Supplementary Figure 6. The funnel plot of Reoperation.

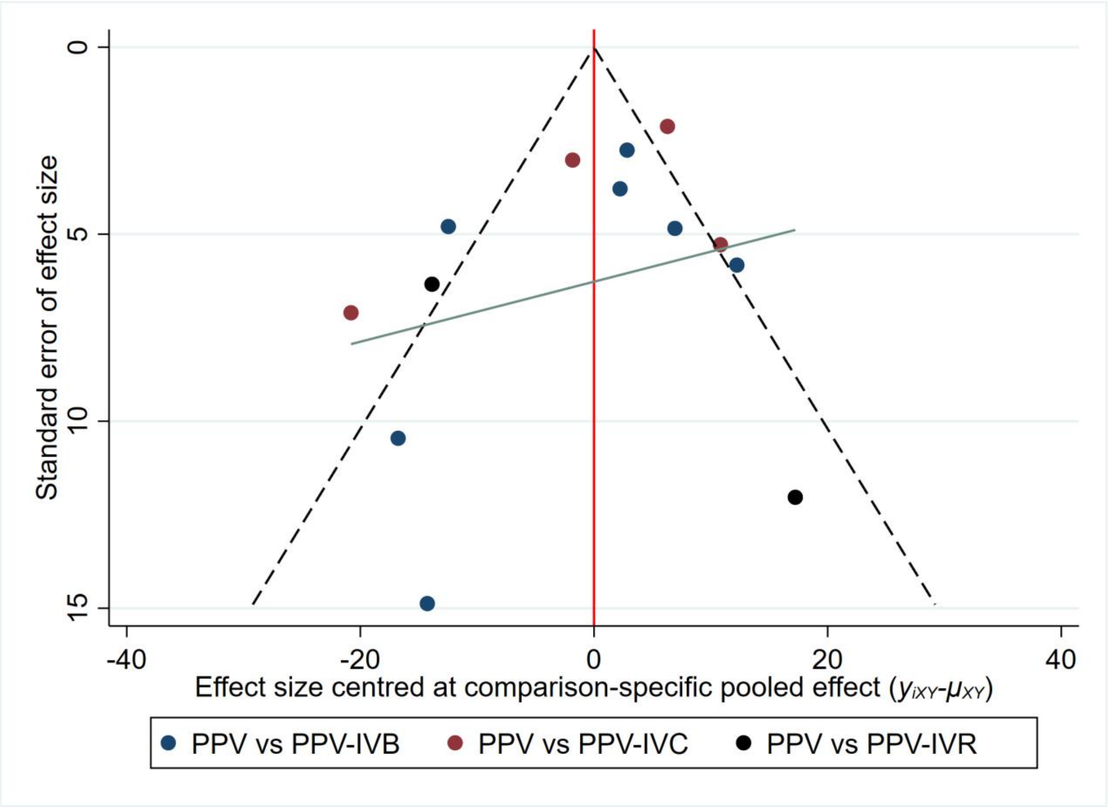

Supplementary Figure 7. The funnel plot of Duration of surgery.

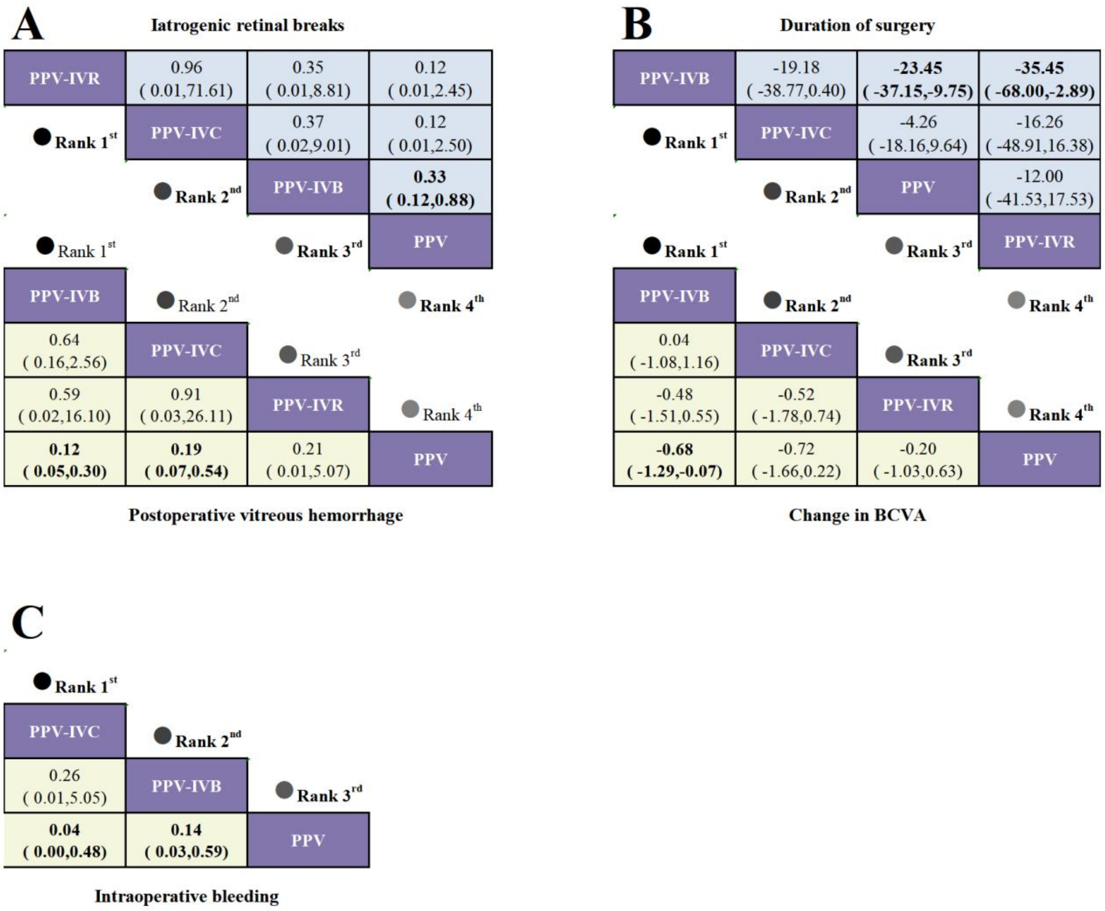

Supplementary Figure 8. Network diagram comparing the safety and efficacy of PPV alone versus PPV combined with different anti-VEGF agents in patients with PDR, restricted to studies with injection timing within the common window (5–7 days) .A: Odds ratios (OR) and 95% confidence intervals (CI) for postoperative vitreous hemorrhage (yellow lower triangular region) and iatrogenic retinal breaks (blue upper triangular region), where OR < 1.00 indicates a lower risk of occurrence. B: Risk ratios and 95% CIs for changes in BCVA (yellow lower triangular region) and Duration of surgery (blue upper triangular region), where a mean difference (MD) < 0 indicates a better clinical outcome. C: Odds ratios and 95% CIs for intraoperative bleeding (yellow lower triangular region).

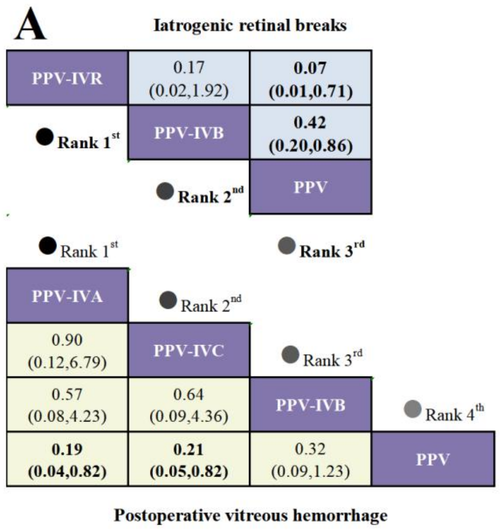

Supplementary Figure 9. Network diagram comparing the safety and efficacy of PPV alone versus PPV combined with different anti-VEGF agents in patients with PDR, restricted to studies with injection timing outside the 5–7 day window (i.e., <5 days or >7 days). A: Odds ratios (OR) and 95% confidence intervals (CI) for postoperative vitreous hemorrhage (yellow lower triangular region) and iatrogenic retinal breaks (blue upper triangular region), where OR < 1.00 indicates a lower risk of occurrence. B: Risk ratios and 95% CIs for changes in BCVA (yellow lower triangular region) and duration of surgery (blue upper triangular region), where a mean difference (MD) < 0 indicates a better clinical outcome.

Supplement 3 Sensitivity Analysis

Table 3.1. Sensitivity Analysis Using Intraoperative bleeding as the Outcome

dropped_id	comparison	effect size	lower confidence interval	upper confidence interval	Connected Network Status
Arevalo JF 2019	PPV-IVB VS PPV	0.08988539	0.024068	0.3356892	1
Arevalo JF 2019	PPV-IVC VS PPV	0.11319895	0.0169644	0.7553483	1
Arevalo JF 2019	PPV-IVR VS PPV	0.03448276	0.0023728	0.5011295	1
Su L 2016	PPV-IVB VS PPV	0.12002844	0.0421831	0.3415308	1
Su L 2016	PPV-IVC VS PPV	0.29824561	0.0284265	3.129139	1
Su L 2016	PPV-IVR VS PPV	0.03448276	0.0029271	0.4062191	1
Ahmadieh H 2009	PPV-IVB VS PPV	0.08360945	0.0252162	0.2772242	1
Ahmadieh H 2009	PPV-IVC VS PPV	0.11491222	0.0197861	0.6673769	1
Ahmadieh H 2009	PPV-IVR VS PPV	0.03448276	0.0028871	0.4118596	1
di Lauro R 2010	PPV-IVB VS PPV	0.17808841	0.06248	0.5076099	1
di Lauro R 2010	PPV-IVC VS PPV	0.11813984	0.0245769	0.5678919	1
di Lauro R 2010	PPV-IVR VS PPV	0.03448276	0.0037899	0.3137431	1
Faisal SM 2018	PPV-IVB VS PPV	0.14197193	0.0416588	0.4838356	1

Faisal SM 2018	PPV-IVC VS PPV	0.11371446	0.0178281	0.725314	1
Faisal SM 2018	PPV-IVR VS PPV	0.03448276	0.0025299	0.4700101	1
Li S 2022	PPV-IVB VS PPV	0.11884702	0.0404912	0.3488318	1
Li S 2022	PPV-IVC VS PPV	0.11436732	0.0189196	0.6913406	1
Rizzo S 2008	PPV-IVB VS PPV	0.12263948	0.0358848	0.4191316	1
Rizzo S 2008	PPV-IVC VS PPV	0.11310294	0.0168014	0.7613838	1
Rizzo S 2008	PPV-IVR VS PPV	0.03448276	0.0023433	0.5074312	1
Yang Z 2023	PPV-IVB VS PPV	0.12002844	0.0421831	0.3415308	1
Yang Z 2023	PPV-IVC VS PPV	0.03571429	0.0026397	0.4832054	1
Yang Z 2023	PPV-IVR VS PPV	0.03448276	0.0029271	0.4062191	1

Table 3.2. Sensitivity Analysis Using Postoperative vitreous hemorrhage as the Outcome Measure

dropped_id	comparison	effect size	lower confidence interval	upper confidence interval	Connected Network Status
Arevalo JF 2019	PPV-IVA VS PPV	0.18644068	0.0615591	0.5646625	1
Arevalo JF 2019	PPV-IVB VS PPV	0.24654981	0.1415408	0.4294649	1
Arevalo JF 2019	PPV-IVC VS PPV	0.24802898	0.1367869	0.4497389	1
Arevalo JF 2019	PPV-IVR VS PPV	0.20967742	0.008517	5.161984	1
El-Batarny AM 2008	PPV-IVA VS PPV	0.18644068	0.0531017	0.6545953	1
El-Batarny AM 2008	PPV-IVB VS PPV	0.31127552	0.1779299	0.5445542	1
El-Batarny AM 2008	PPV-IVC VS PPV	0.25107187	0.130223	0.4840703	1
El-Batarny AM 2008	PPV-IVR VS PPV	0.20967742	0.0080687	5.448797	1
Comyn O 2017	PPV-IVA VS PPV	0.18644068	0.0523225	0.6643438	1
Comyn O 2017	PPV-IVB VS PPV	0.29997405	0.1710251	0.5261476	1
Comyn O 2017	PPV-IVC VS PPV	0.25131636	0.1295497	0.4875343	1
Ahn J 2011	PPV-IVA VS PPV	0.18644068	0.0495497	0.7015205	1
Ahn J 2011	PPV-IVB VS PPV	0.25956046	0.1398333	0.4817998	1
Ahn J 2011	PPV-IVC VS PPV	0.25214109	0.1270761	0.5002917	1
Ahn J 2011	PPV-IVR VS PPV	0.20967742	0.0078513	5.59967	1
Yang X 2016	PPV-IVA VS PPV	0.18644068	0.0486775	0.7140908	1
Yang X 2016	PPV-IVB VS PPV	0.2910031	0.1630308	0.5194283	1
Yang X 2016	PPV-IVC VS PPV	0.27669231	0.1195485	0.640398	1
Yang X 2016	PPV-IVR VS PPV	0.20967742	0.0077949	5.640159	1
Su L 2016	PPV-IVA VS PPV	0.18644068	0.0512079	0.6788037	1
Su L 2016	PPV-IVB VS PPV	0.29708702	0.1684466	0.5239684	1
Su L 2016	PPV-IVC VS PPV	0.26898316	0.1330804	0.5436711	1
Su L 2016	PPV-IVR VS PPV	0.20967742	0.0079551	5.526568	1
Hernández-Da MSE 2010	PPV-IVA VS PPV	0.18644068	0.0484795	0.7170074	1
Hernández-Da MSE 2010	PPV-IVB VS PPV	0.27905793	0.1497306	0.5200897	1
Hernández-Da MSE 2010	PPV-IVC VS PPV	0.25244104	0.1260693	0.5054879	1
Hernández-Da MSE 2010	PPV-IVR VS PPV	0.20967742	0.007782	5.649556	1
Farahvash MS 2011	PPV-IVA VS PPV	0.18644068	0.0494495	0.7029423	1

Farahvash MS 2011	PPV-IVB VS PPV	0.27437074	0.1487781	0.5059836	1
Farahvash MS 2011	PPV-IVC VS PPV	0.25216961	0.126973	0.5008114	1
Farahvash MS 2011	PPV-IVR VS PPV	0.20967742	0.0078449	5.604249	1
Ahmadieh H 2009	PPV-IVA VS PPV	0.18644068	0.0521293	0.6668063	1
Ahmadieh H 2009	PPV-IVB VS PPV	0.31585587	0.1696052	0.5882186	1
Ahmadieh H 2009	PPV-IVC VS PPV	0.25137611	0.1293355	0.488574	1
Ahmadieh H 2009	PPV-IVR VS PPV	0.20967742	0.008011	5.488006	1
di Lauro R 2010	PPV-IVA VS PPV	0.18644068	0.0517294	0.6719607	1
di Lauro R 2010	PPV-IVB VS PPV	0.3101674	0.1699353	0.5661203	1
di Lauro R 2010	PPV-IVC VS PPV	0.25149866	0.1289974	0.490332	1
di Lauro R 2010	PPV-IVR VS PPV	0.20967742	0.0079869	5.504569	1
Manabe A 2015	PPV-IVA VS PPV	0.18644068	0.0539854	0.6438801	1
Manabe A 2015	PPV-IVB VS PPV	0.32023015	0.1824658	0.5620087	1
Manabe A 2015	PPV-IVC VS PPV	0.25078766	0.1309788	0.4801881	1
Manabe A 2015	PPV-IVR VS PPV	0.20967742	0.0081199	5.414429	1
Modarres M 2009	PPV-IVA VS PPV	0.18644068	0.0549489	0.6325899	1
Modarres M 2009	PPV-IVB VS PPV	0.32421977	0.1882526	0.5583906	1
Modarres M 2009	PPV-IVC VS PPV	0.25046921	0.1318125	0.4759399	1
Modarres M 2009	PPV-IVR VS PPV	0.20967742	0.0081745	5.378261	1
Zaman Y 2013	PPV-IVA VS PPV	0.18644068	0.0649235	0.5354012	1
Zaman Y 2013	PPV-IVB VS PPV	0.3944375	0.2656322	0.5857005	1
Zaman Y 2013	PPV-IVC VS PPV	0.24660083	0.1400931	0.4340825	1
Zaman Y 2013	PPV-IVR VS PPV	0.20967742	0.0086718	5.069823	1
Ding Y 2023	PPV-IVA VS PPV	0.18644068	0.0488583	0.7114484	1
Ding Y 2023	PPV-IVB VS PPV	0.29141835	0.1634951	0.5194325	1
Ding Y 2023	PPV-IVC VS PPV	0.26307017	0.1196929	0.5781956	1
Ding Y 2023	PPV-IVR VS PPV	0.20967742	0.0078067	5.631646	1
Qu JF 2023	PPV-IVA VS PPV	0.29997405	0.1710251	0.5261476	1
Qu JF 2023	PPV-IVB VS PPV	0.25131636	0.1295497	0.4875343	1
Qu JF 2023	PPV-IVC VS PPV	0.20967742	0.0080226	5.480096	1
Yang Z 2023	PPV-IVA VS PPV	0.18644068	0.0508132	0.6840761	1
Yang Z 2023	PPV-IVB VS PPV	0.29609716	0.1671122	0.5246387	1
Yang Z 2023	PPV-IVC VS PPV	0.2266055	0.1073331	0.4784176	1
Yang Z 2023	PPV-IVR VS PPV	0.20967742	0.0079308	5.543525	1
Jiang T 2020	PPV-IVA VS PPV	0.18644068	0.0530198	0.6556068	1
Jiang T 2020	PPV-IVB VS PPV	0.30185404	0.1724138	0.5284719	1
Jiang T 2020	PPV-IVC VS PPV	0.2198346	0.1105796	0.437036	1
Jiang T 2020	PPV-IVR VS PPV	0.20967742	0.0080639	5.452044	1
Ren X 2019	PPV-IVA VS PPV	0.18644068	0.0510824	0.6804721	1
Ren X 2019	PPV-IVB VS PPV	0.29677037	0.1681332	0.5238268	1
Ren X 2019	PPV-IVC VS PPV	0.2669308	0.1318439	0.5404275	1
Ren X 2019	PPV-IVR VS PPV	0.20967742	0.0079474	5.531933	1

Table 3.3. Sensitivity Analysis Using Changes in BCVA as the Outcome Measure

dropped_id	comparison	effect size	lower confidence interval	upper confidence interval	Connected Network Status
Arevalo JF 2019	PPV-IVB VS PPV	-0.68035115	-1.425639	0.064937	1
Arevalo JF 2019	PPV-IVC VS PPV	-0.82456795	-2.597978	0.948842	1

Arevalo JF 2019	PPV-IVR VS PPV	-0.51987969	-1.770227	0.7304677	1
El-Batarny AM 2008	PPV-IVB VS PPV	-0.60533381	-1.356898	0.1462304	1
El-Batarny AM 2008	PPV-IVC VS PPV	-0.82456795	-2.632582	0.9834461	1
El-Batarny AM 2008	PPV-IVR VS PPV	-0.51978625	-1.794685	0.7551127	1
Comyn O 2017	PPV-IVB VS PPV	-0.58817391	-1.282058	0.1057106	1
Comyn O 2017	PPV-IVC VS PPV	-0.82456795	-2.629604	0.9804677	1
Comyn O 2017	PPV-IVR VS PPV	-0.62121575	-2.401025	1.158594	1
Su L 2016	PPV-IVB VS PPV	-0.57046976	-1.20488	0.0639404	1
Su L 2016	PPV-IVC VS PPV	-0.52028638	-1.678172	0.6375989	1
Manabe A 2015	PPV-IVB VS PPV	-0.71001197	-1.42114	0.0011159	1
Manabe A 2015	PPV-IVC VS PPV	-0.82456795	-2.517983	0.8688475	1
Manabe A 2015	PPV-IVR VS PPV	-0.52011821	-1.713694	0.6734573	1
Modarres M 2009	PPV-IVB VS PPV	-0.46	-1.148083	0.2358433	1
Modarres M 2009	PPV-IVC VS PPV	-0.82	-2.468215	0.8190794	1
Modarres M 2009	PPV-IVR VS PPV	-0.52	-1.678528	0.6379586	1
Rizzo S 2008	PPV-IVB VS PPV	-0.28	-0.7366335	0.1678201	1
Rizzo S 2008	PPV-IVC VS PPV	-0.82	-1.965688	0.3165523	1
Rizzo S 2008	PPV-IVR VS PPV	-0.52	-1.323855	0.2770654	1
Sohn EH 2012	PPV-IVB VS PPV	-0.54	-1.272578	0.1846203	1
Sohn EH 2012	PPV-IVC VS PPV	-0.82	-2.590398	0.9412624	1
Sohn EH 2012	PPV-IVR VS PPV	-0.5199009	-1.76487	0.7250685	1
Ahn J 2011	PPV-IVB VS PPV	-0.73890832	-1.4015	-0.0763166	1
Ahn J 2011	PPV-IVC VS PPV	-0.82456795	-2.391099	0.7419635	1
Ahn J 2011	PPV-IVR VS PPV	-0.5205748	-1.624045	0.5828958	1
Li S 2022	PPV-IVB VS PPV	-0.58817391	-1.282058	0.1057105	1
Li S 2022	PPV-IVC VS PPV	-0.82456795	-2.629604	0.9804677	1
Li S 2022	PPV-IVR VS PPV	-0.4136393	-2.234505	1.407226	1

Table 3.4. Sensitivity Analysis Using Iatrogenic retinal breaks as the Outcome Measure

dropped_id	comparison	effect size	lower confidence interval	upper confidence interval	Connected Network Status
Arevalo JF 2019	PPV-IVB VS PPV	0.36101455	0.1660535	0.7848765	1
Arevalo JF 2019	PPV-IVC VS PPV	0.11969112	0.0052259	2.741351	1
Arevalo JF 2019	PPV-IVR VS PPV	0.08462679	0.0126926	0.5642416	1
El-Batarny AM 2008	PPV-IVB VS PPV	0.3668961	0.2341859	0.5748114	1
El-Batarny AM 2008	PPV-IVC VS PPV	0.11969112	0.0057304	2.499991	1
El-Batarny AM 2008	PPV-IVR VS PPV	0.08418585	0.0137202	0.516556	1
Comyn O 2017	PPV-IVB VS PPV	0.36745962	0.2383421	0.5665243	1
Comyn O 2017	PPV-IVC VS PPV	0.11969112	0.0057304	2.499991	1
Comyn O 2017	PPV-IVR VS PPV	0.07096774	0.0074459	0.6764063	1
Su L 2016	PPV-IVB VS PPV	0.36745962	0.2383421	0.5665243	1
Su L 2016	PPV-IVC VS PPV	0.08418585	0.0137202	0.5165561	1
Hernández-Da MSE 2010	PPV-IVB VS PPV	0.38197632	0.2442408	0.5973855	1
Hernández-Da MSE 2010	PPV-IVC VS PPV	0.11969112	0.0057304	2.499991	1
Hernández-Da MSE 2010	PPV-IVR VS PPV	0.08418585	0.0137202	0.5165561	1
Ahmadieh H 2009	PPV-IVB VS PPV	0.34826658	0.2243342	0.5406649	1
Ahmadieh H 2009	PPV-IVC VS PPV	0.11969112	0.0057304	2.499991	1

Ahmadieh H 2009	PPV-IVR VS PPV	0.08418585	0.0137202	0.5165561	1
di Lauro R 2010	PPV-IVB VS PPV	0.38534346	0.2478957	0.5990001	1
di Lauro R 2010	PPV-IVC VS PPV	0.11969112	0.0057304	2.499991	1
di Lauro R 2010	PPV-IVR VS PPV	0.08418585	0.0137202	0.5165561	1
Faisal SM 2018	PPV-IVB VS PPV	0.3853445	0.2460724	0.6034418	1
Faisal SM 2018	PPV-IVC VS PPV	0.11969112	0.0057304	2.499991	1
Faisal SM 2018	PPV-IVR VS PPV	0.08418585	0.0137202	0.5165561	1
Li S 2022	PPV-IVB VS PPV	0.36745962	0.2383421	0.5665243	1
Li S 2022	PPV-IVC VS PPV	0.11969112	0.0057304	2.499991	1
Li S 2022	PPV-IVR VS PPV	0.11520737	0.0054263	2.446017	1
Manabe A 2015	PPV-IVB VS PPV	0.32467833	0.2055381	0.5128782	1
Manabe A 2015	PPV-IVC VS PPV	0.11969112	0.0057304	2.499991	1
Manabe A 2015	PPV-IVR VS PPV	0.08418585	0.0137202	0.5165561	1
Rizzo S 2008	PPV-IVB VS PPV	0.37982123	0.2452811	0.5881584	1
Rizzo S 2008	PPV-IVC VS PPV	0.11969112	0.0057304	2.499991	1
Rizzo S 2008	PPV-IVR VS PPV	0.08418585	0.0137202	0.5165561	1

Table 3.5. Sensitivity Analysis Using Reoperation as the Outcome Measure

dropped_id	comparison	effect size	lower confidence interval	upper confidence interval	Connected Network Status
Arevalo JF 2019	PPV-IVB VS PPV	0.43680554	0.2061809	0.9253966	1
Arevalo JF 2019	PPV-IVC VS PPV	1	0.1217997	8.210204	1
El-Batarny AM 2008	PPV-IVB VS PPV	0.38302043	0.2126819	0.6897843	1
El-Batarny AM 2008	PPV-IVC VS PPV	1	0.1217997	8.210203	1
Ahn J 2011	PPV-IVB VS PPV	0.32197799	0.1799321	0.5761608	1
Ahn J 2011	PPV-IVC VS PPV	1	0.1217997	8.210203	1
Hernández-Da MSE 2010	PPV-IVB VS PPV	0.33223768	0.1854373	0.5952517	1
Hernández-Da MSE 2010	PPV-IVC VS PPV	1	0.1217997	8.210204	1
Farahvash MS 2011	PPV-IVB VS PPV	0.34831302	0.1989371	0.6098509	1
Farahvash MS 2011	PPV-IVC VS PPV	1	0.1217997	8.210204	1
di Lauro R 2010	PPV-IVB VS PPV	0.36978025	0.2103852	0.6499385	1
di Lauro R 2010	PPV-IVC VS PPV	1	0.1217997	8.210204	1
Manabe A 2015	PPV-IVB VS PPV	0.3891336	0.220422	0.6869777	1
Manabe A 2015	PPV-IVC VS PPV	1	0.1217997	8.210204	1
Modarres M 2009	PPV-IVB VS PPV	0.35025963	0.2000336	0.6133059	1
Modarres M 2009	PPV-IVC VS PPV	1	0.1217997	8.210204	1
Jiang T 2020	PPV-IVB VS PPV	0.36138742	0.2085789	0.6261461	1

Table 3.6. Sensitivity Analysis Using Duration of surgery as the Outcome Measure

dropped_id	comparison	effect size	lower confidence interval	upper confidence interval	Connected Network Status
Arevalo JF 2019	PPV-IVB VS PPV	-1.0916342	-1.658805	-0.5244633	1
Arevalo JF 2019	PPV-IVC VS PPV	-0.59510703	-1.278052	0.0878384	1
Arevalo JF 2019	PPV-IVR VS PPV	-0.3306224	-1.320611	0.6593658	1

El-Batarny AM 2008	PPV-IVB VS PPV	-0.78801883	-1.26799	-0.308048	1
El-Batarny AM 2008	PPV-IVC VS PPV	-0.58261781	-1.186089	0.020853	1
El-Batarny AM 2008	PPV-IVR VS PPV	-0.3359837	-1.216508	0.5445408	1
Comyn O 2017	PPV-IVB VS PPV	-0.95984925	-1.457311	-0.4623878	1
Comyn O 2017	PPV-IVC VS PPV	-0.59117311	-1.245678	0.0633314	1
Comyn O 2017	PPV-IVR VS PPV	-0.97489356	-2.297354	0.3475666	1
Su L 2016	PPV-IVB VS PPV	-0.96765522	-1.49643	-0.4388801	1
Su L 2016	PPV-IVC VS PPV	-0.44974649	-1.244693	0.3452003	1
Su L 2016	PPV-IVR VS PPV	-0.32988239	-1.338409	0.6786444	1
Hernández-Da MSE 2010	PPV-IVB VS PPV	-0.95636292	-1.545396	-0.36733	1
Hernández-Da MSE 2010	PPV-IVC VS PPV	-0.59957119	-1.319941	0.1207987	1
Hernández-Da MSE 2010	PPV-IVR VS PPV	-0.32865169	-1.370457	0.7131534	1
di Lauro R 2010	PPV-IVB VS PPV	-0.97699224	-1.574516	-0.3794687	1
di Lauro R 2010	PPV-IVC VS PPV	-0.60012337	-1.325576	0.1253295	1
di Lauro R 2010	PPV-IVR VS PPV	-0.32840591	-1.377259	0.7204475	1
Faisal SM 2018	PPV-IVB VS PPV	-0.86454325	-1.423785	-0.305301	1
Faisal SM 2018	PPV-IVC VS PPV	-0.5948746	-1.276078	0.0863283	1
Faisal SM 2018	PPV-IVR VS PPV	-0.33072421	-1.318247	0.6567982	1
Li S 2022	PPV-IVB VS PPV	-0.95984925	-1.457311	-0.4623878	1
Li S 2022	PPV-IVC VS PPV	-0.59117311	-1.245678	0.0633314	1
Li S 2022	PPV-IVR VS PPV	0.35431037	-1.012765	1.721386	1
Manabe A 2015	PPV-IVB VS PPV	-1.0872945	-1.650521	-0.5240681	1
Manabe A 2015	PPV-IVC VS PPV	-0.59509896	-1.277934	0.0877365	1
Manabe A 2015	PPV-IVR VS PPV	-0.33062594	-1.320521	0.6592697	1
Modarres M 2009	PPV-IVB VS PPV	-1.0198179	-1.607708	-0.4319275	1
Modarres M 2009	PPV-IVC VS PPV	-0.59928497	-1.317039	0.1184689	1
Modarres M 2009	PPV-IVR VS PPV	-0.32877892	-1.366987	0.7094296	1
Ding Y 2023	PPV-IVB VS PPV	-0.96160463	-1.465766	-0.457443	1
Ding Y 2023	PPV-IVC VS PPV	-0.85838991	-1.638789	-0.0779904	1
Ding Y 2023	PPV-IVR VS PPV	-0.33178215	-1.294666	0.631102	1
Yang Z 2023	PPV-IVB VS PPV	-0.96232532	-1.469237	-0.4554139	1
Yang Z 2023	PPV-IVC VS PPV	-0.37535649	-1.135241	0.3845275	1
Yang Z 2023	PPV-IVR VS PPV	-0.33155535	-1.299565	0.6364539	1
Ren X 2019	PPV-IVB VS PPV	-0.97045308	-1.51175	-0.4291563	1
Ren X 2019	PPV-IVC VS PPV	-0.71994908	-1.547107	0.1072093	1
Ren X 2019	PPV-IVR VS PPV	-0.32900763	-1.360848	0.702833	1

Supplement 4 Meta-Regression Analysis

Table 4.1. Meta-Regression Analysis of Intraoperative bleeding Using Disease Severity as a Moderator

Interventions		Coefficient	Standard Error	Z-statistic	P>z	lower confidence interval	upper confidence interval
PPV-IVB VS PPV							
	Covariates	-0.8107761	0.8307468	-0.98	0.329	-2.43901	0.8174576
	Constant term	-0.5800426	1.68169	-0.34	0.73	-3.876094	2.716009
PPV-IVC VS PPV							
	Constant term	-2.169681	0.924517	-2.35	0.019	-3.981701	-0.3576613
PPV-IVR VS PPV							
	Constant term	-3.367296	1.303348	-2.58	0.01	-5.921812	-0.8127799

Table 4.2. Meta-Regression Analysis of Postoperative vitreous hemorrhage Using Disease Severity as a Moderator

Interventions		Coefficient	Standard Error	Z-statistic	P>z	lower confidence interval	upper confidence interval
PPV-IVA VS PPV							
	Constant term	-1.679642	0.6193294	-2.71	0.007	-2.893506	-0.4657788
PPV-IVB VS PPV							
	Covariates	-0.4034087	0.4167705	-0.97	0.333	-1.220264	0.4134465
	Constant term	-0.5078414	0.7831424	-0.65	0.517	-2.042772	1.02709
PPV-IVC VS PPV							
	Covariates	-0.6945525	1.213441	-0.57	0.567	-3.072853	1.683748
	Constant term	0.0582833	2.545126	0.02	0.982	-4.930073	5.04664
PPV-IVR VS PPV							
	Constant term	-1.562185	1.653884	-0.94	0.345	-4.803738	1.679368

Table 4.3. Meta-Regression Analysis of Changes in BCVA Using Disease Severity as a Moderator

Interventions		Coefficient	Standard Error	Z-statistic	P>z	lower confidence interval	upper confidence interval
PPV-IVB VS PPV							
	Covariates	0.1480204	0.4783209	0.31	0.757	-0.7894714	1.09
	Constant term	-0.5693667	0.8753646	-0.65	0.515	-2.28505	1.15
PPV-IVC VS PPV							
	Constant term	-0.7200001	0.5506964	-1.31	0.191	-1.799345	0.36
PPV-IVR VS PPV							
	Covariates	-0.02	0.3491416	-0.06	0.954	-0.704305	0.66
	Constant term	-0.18	0.7908657	-0.23	0.82	-1.730068	1.37

Table 4.4. Meta-Regression Analysis of Iatrogenic retinal breaks Using Disease Severity as a Moderator

Interventions		Coefficient	Standard Error	Z-statistic	P>z	lower confidence interval	upper confidence interval
PPV-IVB VS PPV							
	Covariates	-0.0224689	0.4231559	-0.05	0.958	-0.8518392	0.8069013
	Constant term	-0.9738181	0.7072548	-1.38	0.169	-2.360012	0.4123758
PPV-IVC VS PPV							
	Constant term	-2.122841	1.578379	-1.34	0.179	-5.216407	0.9707255
PPV-IVR VS PPV							
	Covariates	-0.2422542	0.9908821	-0.24	0.807	-2.184347	1.699839
	Constant term	-1.918767	2.452815	-0.78	0.434	-6.726196	2.888661

Table 4.5. Meta-Regression Analysis of Reoperation Using Disease Severity as a Moderator

Interventions		Coefficient	Standard Error	Z-statistic	P>z	lower confidence interval	upper confidence interval
PPV-IVB VS PPV							
	Covariates	0.1932782	0.4936807	0.39	0.695	-0.7743183	1.160875
	Constant term	-1.300277	0.7740861	-1.68	0.093	-2.817458	0.2169033
PPV-IVC VS PPV							
	Constant term	-3.47E-11	1.074172	0	1	-2.105339	2.105339

Table 4.6. Meta-Regression Analysis of Duration of surgery Using Disease Severity as a Moderator

Interventions		Coefficient	Standard Error	Z-statistic	P>z	lower confidence interval	upper confidence interval
PPV-IVB VS PPV							
	Covariates	2.363365	7.402659	0.32	0.75	-12.14558	16.87231
	Constant term	-24.62092	15.0673	-1.63	0.102	-54.15227	4.910437
PPV-IVC VS PPV							
	Covariates	9.405734	13.39089	0.7	0.482	-16.83993	35.6514
	Constant term	-30.0972	31.15489	-0.97	0.334	-91.15966	30.96526
PPV-IVR VS PPV							
	Covariates	-15.55	10.40719	-1.49	0.135	-35.94772	4.847721
	Constant term	27.55	25.41994	1.08	0.278	-22.27218	77.37217

Table 4.7. Meta-Regression Analysis of Intraoperative bleeding Using Age as a Moderator

Interventions		Coefficient	Standard Error	Z-statistic	P>z	lower confidence interval	upper confidence interval
PPV-IVB VS PPV							
	Covariates	0.1518888	0.136767	1.11	0.267	-0.1161696	0.4199471
	Constant term	-10.57912	7.642596	-1.38	0.166	-25.55833	4.400095
PPV-IVC VS PPV							
	Covariates	1.434032	1.172698	1.22	0.221	-0.8644146	3.732478
	Constant term	-75.0338	59.60345	-1.26	0.208	-191.8544	41.78681
PPV-IVR VS PPV							
	Constant term	-3.367296	1.219645	-2.76	0.006	-5.757756	-0.9768355

Table 4.8. Meta-Regression Analysis of Postoperative vitreous hemorrhage Using Age as a Moderator

Interventions		Coefficient	Standard Error	Z-statistic	P>z	lower confidence interval	upper confidence interval
PPV-IVA VS PPV							
	Constant term	-1.679642	0.538216	-3.12	0.002	-2.734526	-0.6247583
PPV-IVB VS PPV							
	Covariates	0.1142863	0.0521646	2.19	0.028	0.0120456	0.216527
	Constant term	-7.567524	2.980318	-2.54	0.011	-13.40884	-1.726209
PPV-IVC VS PPV							
	Covariates	0.2329018	0.187704	1.24	0.215	-0.1349914	0.6007949
	Constant term	-13.19521	9.510583	-1.39	0.165	-31.83561	5.445187
PPV-IVR VS PPV							
	Constant term	-1.562185	1.62525	-0.96	0.336	-4.747617	1.623247

Table 4.9. Meta-Regression Analysis of Changes in BCVA Using Age as a Moderator

Interventions		Coefficient	Standard Error	Z-statistic	P>z	lower confidence interval	upper confidence interval
PPV-IVB VS PPV							
	Covariates	0.0399647	0.0359641	1.11	0.266	-0.0305237	0.11
	Constant term	-2.470583	1.963127	-1.26	0.208	-6.318242	1.38
PPV-IVC VS PPV							

	Constant term	-0.7200001	0.4979986	-1.45	0.148	-1.696059	0.26
PPV-IVR VS PPV							
	Covariates	0.0153846	0.2361809	0.07	0.948	-0.4475215	0.48
	Constant term	-1.007693	12.08336	-0.08	0.934	-24.69063	22.68

Table 4.10. Meta-Regression Analysis of Iatrogenic retinal breaks Using Age as a Moderator

Interventions		**Coefficient**	**Standard Error**	**Z-statistic**	**P>z**	**lower confidence interval**	**upper confidence interval**
PPV-IVB VS PPV							
	Covariates	0.0351525	0.0586576	0.6	0.549	-0.0798143	0.1501193
	Constant term	-3.027362	3.336766	-0.91	0.364	-9.567303	3.51258
PPV-IVC VS PPV							
	Constant term	-2.122841	1.561929	-1.36	0.174	-5.184165	0.9384828
PPV-IVR VS PPV							
	Covariates	0.1863495	0.7521274	0.25	0.804	-1.287793	1.660492
	Constant term	-11.94437	38.23732	-0.31	0.755	-86.88813	62.9994

Table 4.11. Meta-Regression Analysis of Reoperation Using Age as a Moderator

Interventions		**Coefficient**	**Standard Error**	**Z-statistic**	**P>z**	**lower confidence interval**	**upper confidence interval**
PPV-IVB VS PPV							
	Covariates	-0.0075303	0.053703	-0.14	0.888	-0.1127862	0.0977255
	Constant term	-0.5932453	3.040725	-0.2	0.845	-6.552957	5.366467
PPV-IVC VS PPV							
	Constant term	-2.14E-10	1.074172	0	1	-2.105339	2.105339

Table 4.12. Meta-Regression Analysis of Duration of surgery Using Age as a Moderator

Interventions		Coefficient	Standard Error	Z-statistic	P>z	lower confidence interval	upper confidence interval
PPV-IVB VS PPV							
	Covariates	1.158332	0.7781793	1.49	0.137	-0.3668718	2.683535
	Constant term	-83.06937	42.776	-1.94	0.052	-166.9088	0.7700383
PPV-IVC VS PPV							
	Covariates	2.618656	6.447161	0.41	0.685	-10.01755	15.25486
	Constant term	-142.4901	330.2704	-0.43	0.666	-789.8082	504.828
PPV-IVR VS PPV							
	Covariates	11.96154	7.22585	1.66	0.098	-2.200861	26.12395
	Constant term	-615.9811	367.2884	-1.68	0.094	-1335.853	103.8909

Table 4.13. Meta-Regression Analysis of Intraoperative bleeding Using Injection Timing as a Moderator

Interventions		Coefficient	Standard Error	Z-statistic	P>z	lower confidence interval	upper confidence interval
PPV-IVB VS PPV							
	Covariates	-0.3339158	0.4564777	-0.73	0.464	-1.228596	0.560764
	Constant term	-0.1090032	2.845815	-0.04	0.969	-5.686697	5.468691
PPV-IVC VS PPV							
	Constant term	-2.179776	0.9746031	-2.24	0.025	-4.089963	-0.2695892
PPV-IVR VS PPV							
	Constant term	-3.367296	1.37431	-2.45	0.014	-6.060894	-0.6736973

Table 4.14. Meta-Regression Analysis of Postoperative vitreous hemorrhage Using Injection Timing as a Moderator

Interventions		Coefficient	Standard Error	Z-statistic	P>z	lower confidence interval	upper confidence interval
PPV-IVA VS PPV							
	Constant term	-1.679642	0.7060118	-2.38	0.017	-3.0634	-0.2958844

PPV-IVB VS PPV							
	Covariates	-0.0570684	0.154708	-0.37	0.712	-0.3602904	0.2461536
	Constant term	-0.9700014	0.8567784	-1.13	0.258	-2.649256	0.7092533
PPV-IVC VS PPV							
	Covariates	0.0430967	0.2059314	0.21	0.834	-0.3605215	0.446715
	Constant term	-1.700988	1.194023	-1.42	0.154	-4.041229	0.6392537
PPV-IVR VS PPV							
	Constant term	-1.562185	1.688258	-0.93	0.355	-4.87111	1.74674

Table 4.15. Meta-Regression Analysis of Changes in BCVA Using Injection Timing as a Moderator

Interventions		**Coefficient**	**Standard Error**	**Z-statistic**	**P>z**	**lower confidence interval**	**upper confidence interval**
PPV-IVB VS PPV							
	Covariates	-0.117869	0.1025632	-1.15	0.25	-0.3188893	0.0831512
	Constant term	0.2048641	0.4686557	0.44	0.662	-0.7136842	1.123412
PPV-IVC VS PPV							
	Constant term	-0.7200001	0.4876891	-1.48	0.14	-1.675853	0.2358531
PPV-IVR VS PPV							
	Covariates	0.01	0.1493209	0.07	0.947	-0.2826636	0.3026636
	Constant term	-0.27	0.7916083	-0.34	0.733	-1.821524	1.281524

Table 4.16. Meta-Regression Analysis of Iatrogenic retinal breaks Using Injection Timing as a Moderator

Interventions		**Coefficient**	**Standard Error**	**Z-statistic**	**P>z**	**lower confidence interval**	**upper confidence interval**
PPV-IVB VS PPV							
	Covariates	-0.1587978	0.1332168	-1.19	0.233	-0.419898	0.1023024
	Constant term	-0.3415216	0.5958123	-0.57	0.567	-1.509292	0.8262491

PPV-IVC VS PPV							
	Constant term	-2.122841	1.550576	-1.37	0.171	-5.161913	0.9162313
PPV-IVR VS PPV							
	Covariates	0.1211271	0.4843422	0.25	0.803	-0.8281662	1.07042
	Constant term	-3.008911	2.327919	-1.29	0.196	-7.571548	1.553726

Table 4.17. Meta-Regression Analysis of Reoperation Using Injection Timing as a Moderator

Interventions		Coefficient	Standard Error	Z-statistic	P>z	lower confidence interval	upper confidence interval
PPV-IVB VS PPV							
	Covariates	0.0421189	0.1814129	0.23	0.816	-0.3134439	0.3976816
	Constant term	-1.194957	0.8129245	-1.47	0.142	-2.78826	0.3983454

Table 4.18. Meta-Regression Analysis of Duration of surgery Using Injection Timing as a Moderator

Interventions		**Coefficient**	**Standard Error**	**Z-statistic**	**P>z**	**lower confidence interval**	**upper confidence interval**
PPV-IVB VS PPV							
	Covariates	-0.5686415	2.146543	-0.26	0.791	-4.775789	3.638506
	Constant term	-17.36321	11.33703	-1.53	0.126	-39.58337	4.856953
PPV-IVC VS PPV							
	Constant term	-11.26396	7.014182	-1.61	0.108	-25.0115	2.483583
PPV-IVR VS PPV							
	Covariates	7.775	5.150139	1.51	0.131	-2.319087	17.86909
	Constant term	-42.425	25.26682	-1.68	0.093	-91.94706	7.097062

Supplement 5 GRADE Assessment of Evidence Certainty

Table 5.1. Intraoperative bleeding

Comparison	Number of studies	Within-study bias	Reporting bias	Indirectness	Imprecision	Heterogeneity	Incoherence	Confidence rating	Reason(s) for downgrading
PPV:PPV-IVB	5	No concerns	Low risk	No concerns	No concerns	Some concerns	Some concerns	Low	["Heterogeneity","Incoherence"]
PPV:PPV-IVC	2	Some concerns	Low risk	No concerns	No concerns	Some concerns	Some concerns	Very low	["Within-study bias","Heterogeneity","Incoherence"]
PPV:PPV-IVR	1	No concerns	Low risk	No concerns	No concerns	Some concerns	Some concerns	Low	["Heterogeneity","Incoherence"]
PPV-IVB:PPV-IVC	0	No concerns	Low risk	No concerns	Some concerns	No concerns	Some concerns	Low	["Imprecision","Incoherence"]
PPV-IVB:PPV-IVR	0	No concerns	Low risk	No concerns	Some concerns	No concerns	Some concerns	Low	["Imprecision","Incoherence"]
PPV-IVC:PPV-IVR	0	No concerns	Low risk	No concerns	Some concerns	No concerns	Some concerns	Low	["Imprecision","Incoherence"]

Table Notes: Within-study bias: The majority of the primary evidence in the comparison is derived from studies at risk of having "some concerns" regarding their quality. Imprecision: The 95% confidence interval is too wide and spans the predefined clinically significant effect threshold. Incoherence: As the outcome measure is an open-ended structure, there are no results for indirect comparisons; therefore, the grade is downgraded by one level. Heterogeneity: The prediction interval extends into clinically significant effects in both directions.

Table 5.2. Postoperative vitreous hemorrhage

Comparison	Number of studies	Within-study bias	Reporting bias	Indirectness	Imprecision	Heterogeneity	Incoherence	Confidence rating	Reason(s) for downgrading
PPV:PPV-IVA	1	No concerns	Low risk	No concerns	No concerns	No concerns	Some concerns	Moderate	["Incoherence"]
PPV:PPV-IVB	10	No concerns	Low risk	No concerns	No concerns	No concerns	Some concerns	Moderate	["Incoherence"]
PPV:PPV-IVC	6	No concerns	Low risk	No concerns	No concerns	No concerns	Some concerns	Moderate	["Incoherence"]
PPV:PPV-IVR	1	No concerns	Low risk	No concerns	Some concerns	No concerns	Some concerns	Low	["Imprecision","Incoherence"]

PPV-IVA:PPV-IVB	0	No concerns	Low risk	No concerns	Some concerns	No concerns	Some concerns	Low	["Imprecision","Incoherence"]
PPV-IVA:PPV-IVC	0	No concerns	Low risk	No concerns	Some concerns	No concerns	Some concerns	Low	["Imprecision","Incoherence"]
PPV-IVA:PPV-IVR	0	No concerns	Low risk	No concerns	Some concerns	No concerns	Some concerns	Low	["Imprecision","Incoherence"]
PPV-IVB:PPV-IVC	0	No concerns	Low risk	No concerns	Some concerns	No concerns	Some concerns	Low	["Imprecision","Incoherence"]
PPV-IVB:PPV-IVR	0	No concerns	Low risk	No concerns	Some concerns	No concerns	Some concerns	Low	["Imprecision","Incoherence"]
PPV-IVC:PPV-IVR	0	No concerns	Low risk	No concerns	Some concerns	No concerns	Some concerns	Low	["Imprecision","Incoherence"]

Table 5.3. Changes in BCVA

Comparison	Number of studies	Within-study bias	Reporting bias	Indirectness	Imprecision	Heterogeneity	Incoherence	Confidence rating	Reason(s) for downgrading
PPV:PPV-IVB	7	No concerns	Low risk	No concerns	No concerns	Some concerns	Some concerns	Low	["Heterogeneity","Incoherence"]
PPV:PPV-IVC	1	No concerns	Low risk	No concerns	Some concerns	No concerns	Some concerns	Low	["Imprecision","Incoherence"]
PPV:PPV-IVR	2	No concerns	Low risk	No concerns	No concerns	No concerns	Some concerns	Moderate	["Incoherence"]
PPV-IVB:PPV-IVC	0	No concerns	Low risk	No concerns	Some concerns	No concerns	Some concerns	Low	["Imprecision","Incoherence"]
PPV-IVB:PPV-IVR	0	No concerns	Low risk	No concerns	Some concerns	No concerns	Some concerns	Low	["Imprecision","Incoherence"]
PPV-IVC:PPV-IVR	0	No concerns	Low risk	No concerns	Some concerns	No concerns	Some concerns	Low	["Imprecision","Incoherence"]

Table 5.4. Iatrogenic retinal breaks

Comparison	Number of studies	Within-study bias	Reporting bias	Indirectness	Imprecision	Heterogeneity	Incoherence	Confidence rating	Reason(s) for downgrading
PPV:PPV-IVB	8	No concerns	Low risk	No concerns	No concerns	No concerns	Some concerns	Moderate	["Incoherence"]
PPV:PPV-IVC	1	No concerns	Low risk	No concerns	Some concerns	No concerns	Some concerns	Low	["Imprecision","Incoherence"]
PPV:PPV-IVR	2	No concerns	Low risk	No concerns	No concerns	No concerns	Some concerns	Moderate	["Incoherence"]
PPV-IVB:PPV-IVC	0	No concerns	Low risk	No concerns	Some concerns	No concerns	Some concerns	Low	["Imprecision","Incoherence"]
PPV-IVB:PPV-IVR	0	No concerns	Low risk	No concerns	Some concerns	No concerns	Some concerns	Low	["Imprecision","Incoherence"]
PPV-IVC:PPV-IVR	0	No concerns	Low risk	No concerns	Some concerns	No concerns	Some concerns	Low	["Imprecision","Incoherence"]

Table 5.5. Reoperation

Comparison	Number of studies	Within-study bias	Reporting bias	Indirectness	Imprecision	Heterogeneity	Incoherence	Confidence rating	Reason(s) for downgrading
PPV:PPV-IVB	8	No concerns	Low risk	No concerns	No concerns	No concerns	Some concerns	Moderate	["Incoherence"]
PPV:PPV-IVC	1	No concerns	Low risk	No concerns	Some concerns	No concerns	Some concerns	Low	["Imprecision","Incoherence"]
PPV-IVB:PPV-IVC	0	No concerns	Low risk	No concerns	Some concerns	No concerns	Some concerns	Low	["Imprecision","Incoherence"]

Table 5.6. Duration of surgery

Comparison	Number of studies	Within-study bias	Reporting bias	Indirectness	Imprecision	Heterogeneity	Incoherence	Confidence rating	Reason(s) for downgrading
PPV:PPV-IVB	7	No concerns	Low risk	No concerns	No concerns	Some	Some	Low	["Heterogeneity","Inco

						concerns	concerns		herence"]
PPV:PPV-IVC	4	No concerns	Low risk	No concerns	No concerns	Some concerns	Some concerns	Low	["Heterogeneity","Incoherence"]
PPV:PPV-IVR	2	No concerns	Low risk	No concerns	Some concerns	No concerns	Some concerns	Low	["Imprecision","Incoherence"]
PPV-IVB:PPV-IVC	0	No concerns	Low risk	No concerns	No concerns	Some concerns	Some concerns	Low	["Heterogeneity","Incoherence"]
PPV-IVB:PPV-IVR	0	No concerns	Low risk	No concerns	Some concerns	Some concerns	Some concerns	Very low	["Imprecision","Heterogeneity","Incoherence"]
PPV-IVC:PPV-IVR	0	No concerns	Low risk	No concerns	Some concerns	No concerns	Some concerns	Low	["Imprecision","Incoherence"]
